# Illuminating nature’s beauty: modular, scalable and low-cost LED dome illumination system using 3D-printing technology

**DOI:** 10.1038/s41598-020-69075-y

**Published:** 2020-07-22

**Authors:** Fabian Bäumler, Alexander Koehnsen, Halvor T. Tramsen, Stanislav N. Gorb, Sebastian Büsse

**Affiliations:** Functional Morphology and Biomechanics, Institute of Zoology, Kiel University, Kiel, Germany

**Keywords:** Biological techniques, Imaging, Optical imaging, Zoology

## Abstract

Presenting your research in the proper light can be exceptionally challenging. Meanwhile, dome illumination systems became a standard for micro- and macrophotography in taxonomy, morphology, systematics and especially important in natural history collections. However, proper illumination systems are either expensive and/or laborious to use. Nowadays, 3D-printing technology revolutionizes lab-life and will soon find its way into most people’s everyday life. Consequently, fused deposition modelling printers become more and more available, with online services offering personalized printing options. Here, we present a 3D-printed, scalable, low-cost and modular LED illumination dome system for scientific micro- and macrophotography. We provide stereolithography ('.stl') files and print settings, as well as a complete list of necessary components required for the construction of three differently sized domes. Additionally, we included an optional iris diaphragm and a sliding table, to arrange the object of desire inside the dome. The dome can be easily scaled and modified by adding customized parts, allowing you to always present your research object in the best light.

## Introduction

Depicting the complexity and diversity of biological objects is still an important, but non-trivial task. Despite modern technology, like confocal laser scanning microscopy (CLSM; cf.^1–4^) or micro computed tomography (µCT; cf.^5–8^), have enabled a renaissance of morphology^9^—photography is still indispensable in many cases. Particularly taxonomy, morphology and systematics studies (cf.^10–16^) strongly rely on and benefit from expressive images of the species in focus. Additionally, in natural history collections—where minimizing specimen digitalization efforts has high priority^17^—digitally available specimens prevent the risk of loss or damage to valuable original museums specimens and diminish the need of expensive journeys. Comprehensive attempts have been made in developing techniques to digitize museums specimens, using semi-automated photogrammetry as a morphometric approach^18^.

In all cases, highly detailed images are necessary to display and optimally present key features, diagnostic characters and a detailed habitus view^19^. However, the necessary equipment to generate meaningful, high quality images is often expensive^20^. In order to achieve detailed and compelling scientific images of an investigated object, proper illumination is of particular importance^19,21–24^. Therefore, especially when using higher magnifications, high intensity diffuse light is needed to reveal microscopic structures (e.g. setae or cuticle formations) in detail, which might lead to unintentional over exposition of other structures^19^. ‘Hard lighting’, caused by a small and undiffuse light source, also leads to artefacts, such as glare and flaring, impacting image quality and likely distorting features of the photographed object^21,22^. To minimize these artefacts, Buffington et al.^21,22^ showed that a hemispherical dome, to scatter light, is highly effective for proper illumination. Using light emitted from the base of the dome, the investigated object is only illuminated by light scattered from the inner dome surface, leading to diffuse, soft illumination^19^. Yet, the setup described by Buffington et al.^19,24^ requires time and experience. There are also commercial solutions available, but these tend to be expensive and one dome size is often insufficient for proper illumination of differently sized objects^19,24^. On the one hand, an object can be simply too large to fit inside a certain sized dome and on the other hand, if a dome is too large for an object, the reduction of light intensity caused by the distance between the reflecting inner dome surface and the object results in a loss of resolution of fine structures^19^. As described by the inverse square law, the intensity of a light source decreases with distance, therefore larger domes need more light emission to achieve the same lighting intensity. This means that either more light is necessary, or small-scale details will be lost due to the effective resolution of the camera^19^. As mentioned earlier, the most promising approach is a hemispherical dome illumination system. Dipping the object of desire in soft and diffuse light, prevents direct light exposition and therefore, sharp shadows and specular highlighting effects^19,22,24^. Here, such a hemispherical dome is used in combination with a light source (LED-rings) at the base of the dome, to create a soft, uniformly scattered and diffuse illumination^19,22,24^.

**Figure 1:**
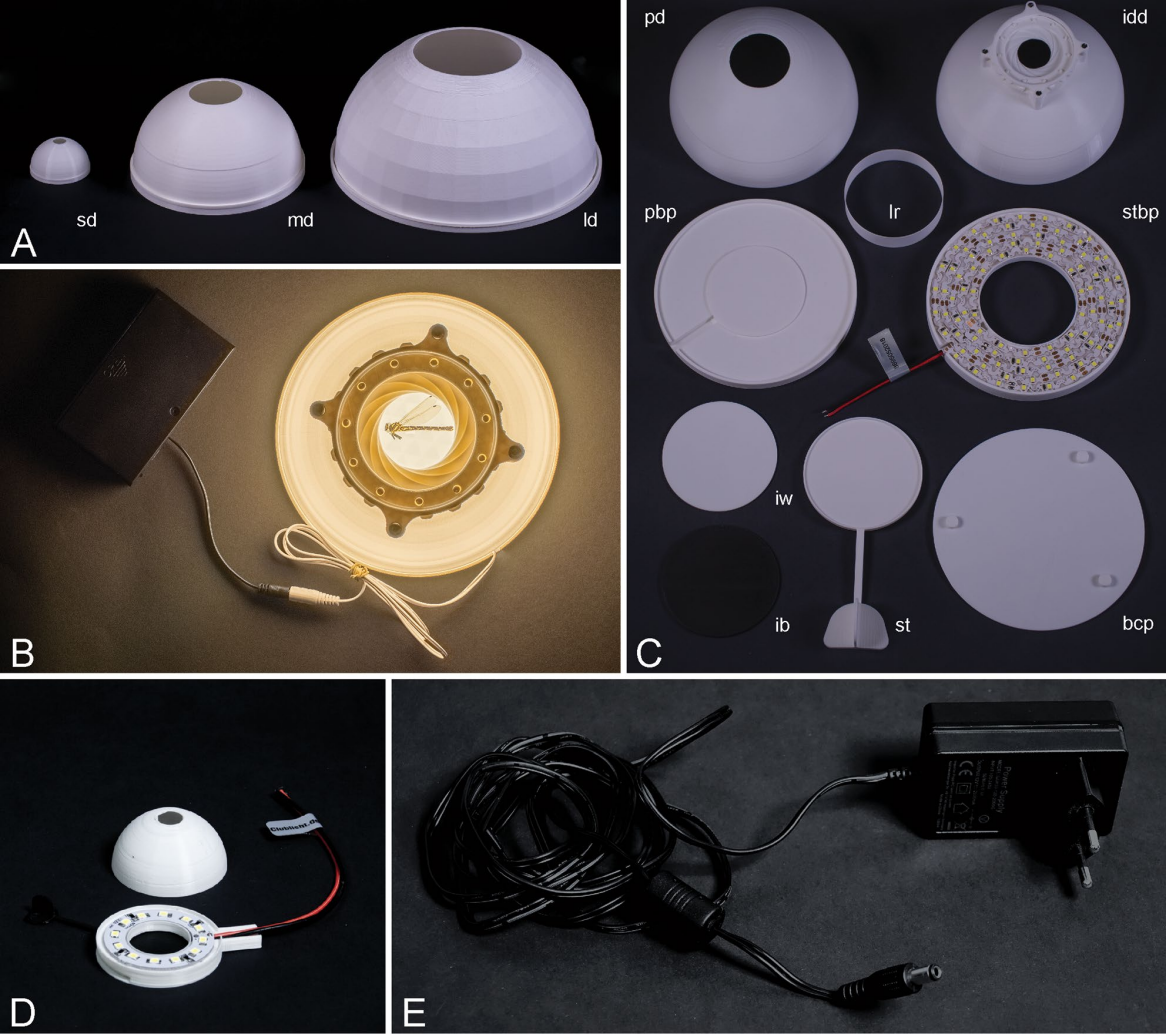
Dome parts: (**A**) small sized dome (sd), medium sized dome (md), large sized dome (ld); (**B**) dome powered by battery box; (**C**) bottom cover plate (bcp), inlays black and white (ib, iw), iris diaphragm dome (idd), light shielding ring (lr), plain baseplate (pb), plain dome (pd), sliding table (st), sliding table baseplate (stbp); (**D**) small dome with LED ring; (**E**) power supply unit.

Today, additive manufacturing (3D-printing) is becoming more and more accessible. Due to both, low machine and material costs, especially fused deposition modelling (FDM) printers are increasingly popular in biology labs^25–29^ and even field works^30,31^. The computer assisted design process facilitates an easy scaling of components, enabling the adaptation of the system to cover a wide range of differently sized samples (as suggested in^24^). We expand on our precursors ^19,22,24^ in presenting a scalable, modular 3D-printed dome for micro- and macrophotography, with diffuse LED-lighting. The innovations of our system are: (1) an optional sliding table to arrange and re-arrange the object of desire while inside the dome (Fig. 1C, st) (2) an optional iris diaphragm to maximize light yield (Fig. 1C, idd), (3) a battery source to even use the system in the field as well as (4) the construction method and material of the system. Our system, therefore, represents an improvement of earlier approaches, especially in terms of quality, durability and reproducibility making it the first system that can seriously compete with commercial solutions. We provide the Blender file, stereolithography (‘.stl’) files and print settings, as well as a complete list of necessary components required for the construction of domes in three different sizes (Fig. 1A). All files are ready to print and can be quickly and easily assembled—leaving you with minimized effort, costs and construction time.

## Materials and methods

### Specimens and objects

**Figure 2:**
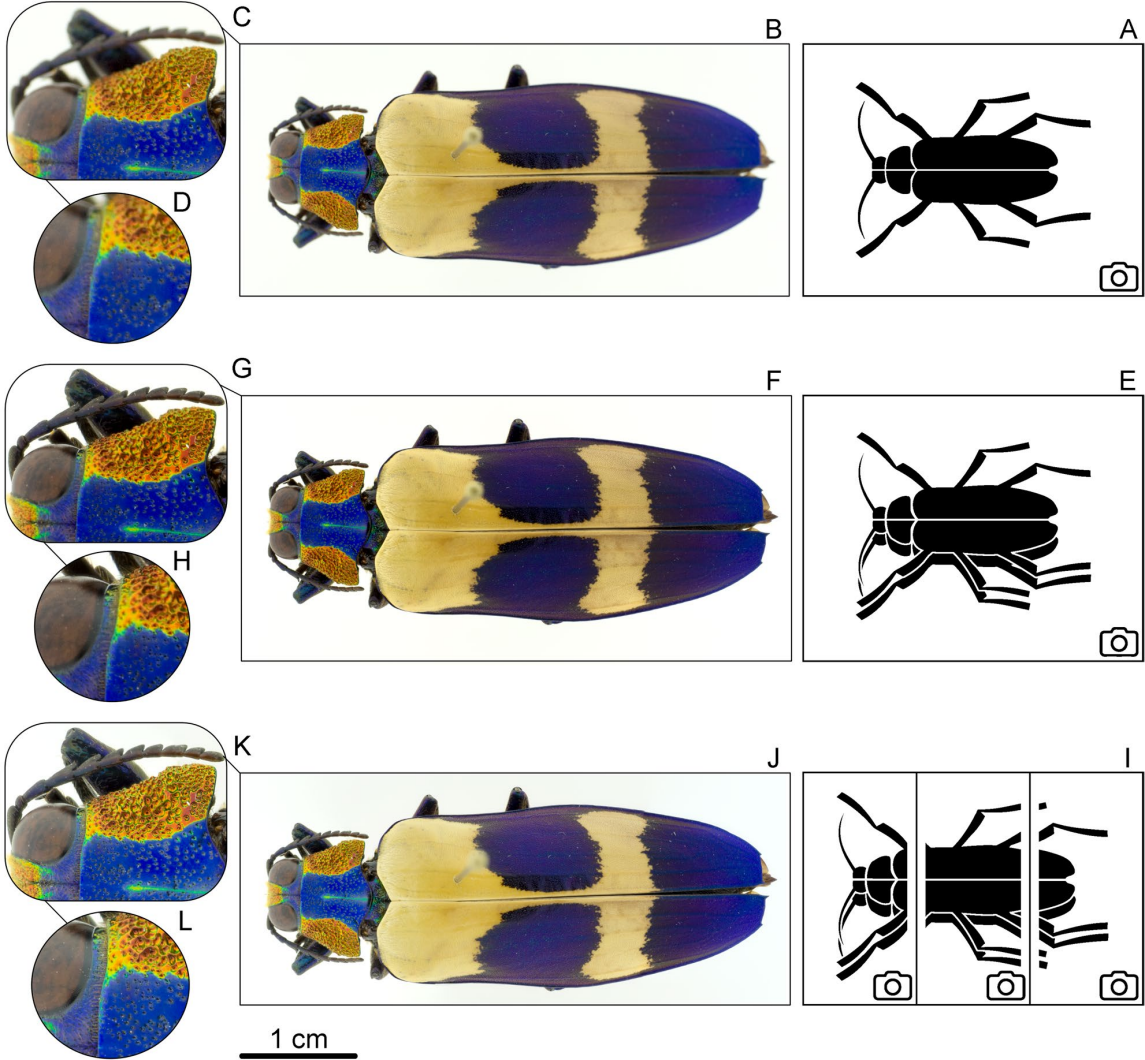
The beetle *Chrysochroa buqueti* (Coleoptera: Buprestidae), dorsal overview and two further magnifications respectively, left is anterior: (**B**–**D**) single image (as pictogram in **A**); (**F**–**H**) 5 focus-layer images stacked (as pictogram in **E**); (**J**–**L**) 3 images stacked, 5 focus layer-images stitched respectively (as pictogram in **I**).

Specimens and objects were adhered to pins, placed flat on a glass disk or an upside-down glass Petri dish on the table of the dome. For specimens in fluids (water, ethanol, glycerine etc.), we used a glass Petri dish as well. A complete list of all specimens and other research objects used for our images can be found in Supplementary Information 1. All necessary permits for using museum specimens in our study were obtained from the Zoological Museum in Kiel. The objects of desire were photographed using an Olympus OMD 10mkII digital camera (Olympus K.K., Tokyo, Japan), equipped with a Leica 45 mm macro lens (Leica, Wetzlar, Germany) with the large dome. Images from different focus layers were combined in Affinity Photo (Version 1.7, Serif Europe Ltd. Apple, https://affinity.serif.com), using the 'focus-stacking' function to generate a single combined-focus image and in some cases the 'panorama' function to combine multiple focus-stacks (Fig. 2). The dome illumination system used here for comparison, was the paper dome designed by Kawada et al.^24^.

### Blender

The open source creation suite Blender (V2.8 Blender Foundation, The Netherlands, www.blender.org) was used to design all parts for the light dome. Parts were individually exported as '.stl' files for further processing. For the iris diaphragm, the mechanical iris model by “Pedro Sousa of Sketchpunk Labs” was used (https://www.thingiverse.com/thing:773759), which is distributed under an Attribution-Non-commercial 3.0 Unported (CC BY-NC 3.0) license. The original model was modified to fit the dome.

### Fused deposition modelling (FDM) 3D printing

All parts were manufactured using a Prusa i3 MK3S (Prusa Research s.r.o., Prague, Czech Republic) with black (for one of the inlays) and white (for all other parts) coloured polylactic acid (PLA) filament (Prusa Research s.r.o., Prague, Czech Republic). Although PLA can be obtained in a very broad range of colours, we recommend using white PLA, to maximize reflection and avoiding an unwanted change of the colour of the reflected light. Prior to printing, the software Slic3r PE (Version 1.2, Prusa Research s.r.o., Prague, Czech Republic) was used to assemble parts into printing batches, and convert the 'stl.' files into '.gcode' files readable by the printer. We printed at an extruder temperature of 215 °C, and a bed temperature of 60 °C. The layer height was set to 0.2 mm and infill to 15%. Furthermore, we used “only on build plate” support structures to print the dome and the “spiral-vase” mode for the light-shielding ring. All other printing settings were left on default state of the PrusaSlicer for Generic PLA Filament but can be found in Supplementary Information 2. Print settings may vary between different printers (even of the same model) and filament types. Therefore, adjusting the parameters to your printer may be necessary.

### Ethics

Animal specimens used in this study are from museum collection only and no ethical statement is necessary.

## Results

**Figure 3:**
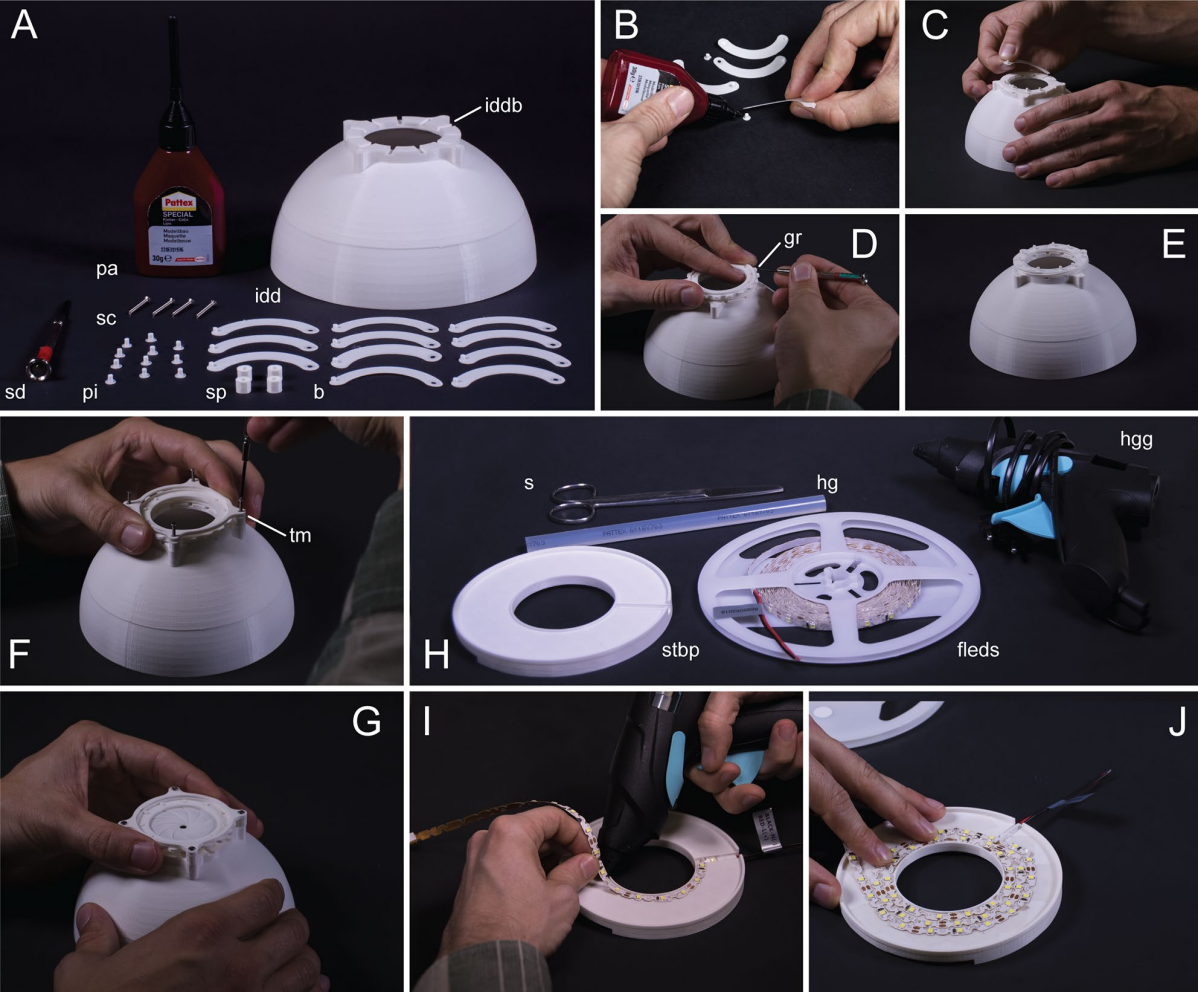
Iris diaphragm assemblage and lighting installation: (**A**) overview of printed parts and needed materials, blades (b), iris diaphragm dome (idd) with baseplate (iddbp), pattex (pa), pins (pi), spacers (sp), screwdriver (sd), screws (sc); (**B**) gluing pins (p) to blades (b); (**C**) adjusting blades on baseplate; (**D**) plugging the gearing (gr) onto the blade pins; (**E**) all blades assembled; (**F**) arranging spacers and screwing top mount (tm) on top; (**G**) finished iris diaphragm; (**H**) materials for lighting installation, scissors (s), hot glue gun (hgg) and hot glue (hg), flexible LED strip (fleds), sliding table baseplate (stbp); (**I**) preparing the sliding table baseplate with hot glue; (**J**) affixing flexible LED strip to sliding table baseplate.

All necessary materials for assembling the dome in three different sizes as well as all accessories are listed in the Supplementary Informations 3 and 4. The procedure for assembling all parts is shown in Fig. 3, using the medium sized dome as an example. The assembly of all dome sizes follows the same method. We provide the Blender file (Supplementary Information 5) as well as all 'stl.' files (see data accessibility) for three different sizes—small for a light microscope, medium for a stereomicroscope and large for a e.g. single-lens reflex camera—but all files can be freely scaled, adjusted and modified by the user, if necessary.

### Assembly of printed parts

Initially, all parts for the respective dome have to be printed (for a list of parts, see Supplementary Information 3). The plain dome requires no further assembly except lighting installation (see “Lighting installation”). To include the optional sliding table, a different base plate, as well as the additional bottom cover plate, has to be printed (Fig. 1C, stbp, bcp). The bottom cover plate holds the sliding table in place and could be glued for more stability. Furthermore, an adjusted light shielding ring, that is placed in the middle of the baseplate of the dome, surrounding the object (Fig. 1C, lr), belongs to every dome size.

### Iris diaphragm

First of all, the blade pins have to be glued into the blade holes (mind the orientation, Fig. 3B), using Pattex modelling adhesive (or comparable). After polymerization, the blades can be placed into the prepared grooves of the iris diaphragm base plate, on top of the dome—with the glued pins facing upwards (as shown in Fig. 3C). Next, the gear ring has to be adjusted and plugged onto the blades, so that the pins pinch through the gear ring’s holes (Fig. 3D,E). For the latter two tasks, using a little screwdriver to adjust the orientation of the blades is extremely handy. Finally, the spacers are placed on top of the four screw-holes in the baseplate and the top mount has to be screwed on top (Fig. 3F,G)—with flat surface facing upwards. Notice, that overtightened screws may impact the ease of movement.

### Lighting installation

We used flexible LED strips in 'neutral white' (V-Tac, 6,400 K) for the medium and large sized dome and a single SMD LED ring (HexaCube, 6,000 K, 40 mm) for the small sized dome. Furthermore, we tried different setups beforehand, which were insufficient in our opinion—a combination of three different sized angel eyes SMD LED rings (HexaCube, 6,000 K) for medium and large sized dome^24^ and single SMD LED’s (WÜRTH, 6,000 K) for the small sized dome.

### Flexible LED strip

We used a flexible adhesive LED strip for lighting (warm: FlexLED 3D Basisset, Paulmann, 3,000 K; neutral: V-Tac, 6,400 K). The LED strip is affixed to the baseplate in a spiral pattern and cut to a suitable length (Fig. 3H,J). The adhesive of the strip is not sufficient to hold it in position, so additional preparation with hot glue or similar adhesives is recommended (Fig. 3I,J). To achieve proper illumination even in the small sized dome, we used a single SMD LED ring with a diameter of 40 mm (Fig. 1d).

The used power supply unit (PSU) requires a DC coaxial connector [5.5 mm outer and 2.1 mm inner diameter, negative barrel, (IEC 60130-10:1971 Type A)]. If LED strips with pre-soldered connector (Fig. 1E) are not available, the connector has to be soldered to the wire that is attached to the LED ring/strip. In this case, we used a connector in combination with a switch for convenience. Adding a switch also allows for protection of the soldering joints inside the switch casing. The dome system is powered by a 12 V DC PSU (see Supplementary Information 3). Generally, it is possible to use any 12 V battery box (e.g. 8 × 1.5 V AA Alkaline Batteries, Fig. 1B) to power the dome.

### Angel eyes LED rings

The assembling process is described by Kawada and Buffington^24^, involving angel eyes SMD LED rings in 3 different diameters (for the medium sized dome: Hexacube, 80 mm/100 mm/120 mm, neutral white, 6,000 K) and several soldering steps.

### Single SMD LED’s

We tried to design our own LED setup to assemble as many LED’s as possible in the small sized dome, by connecting single SMD LED’s with enamelled copper wire and the needed resistors. However, this procedure is not beneficial and too time consuming; consequently, we used a single SMD LED ring instead.

### Images

**Figure 4:**
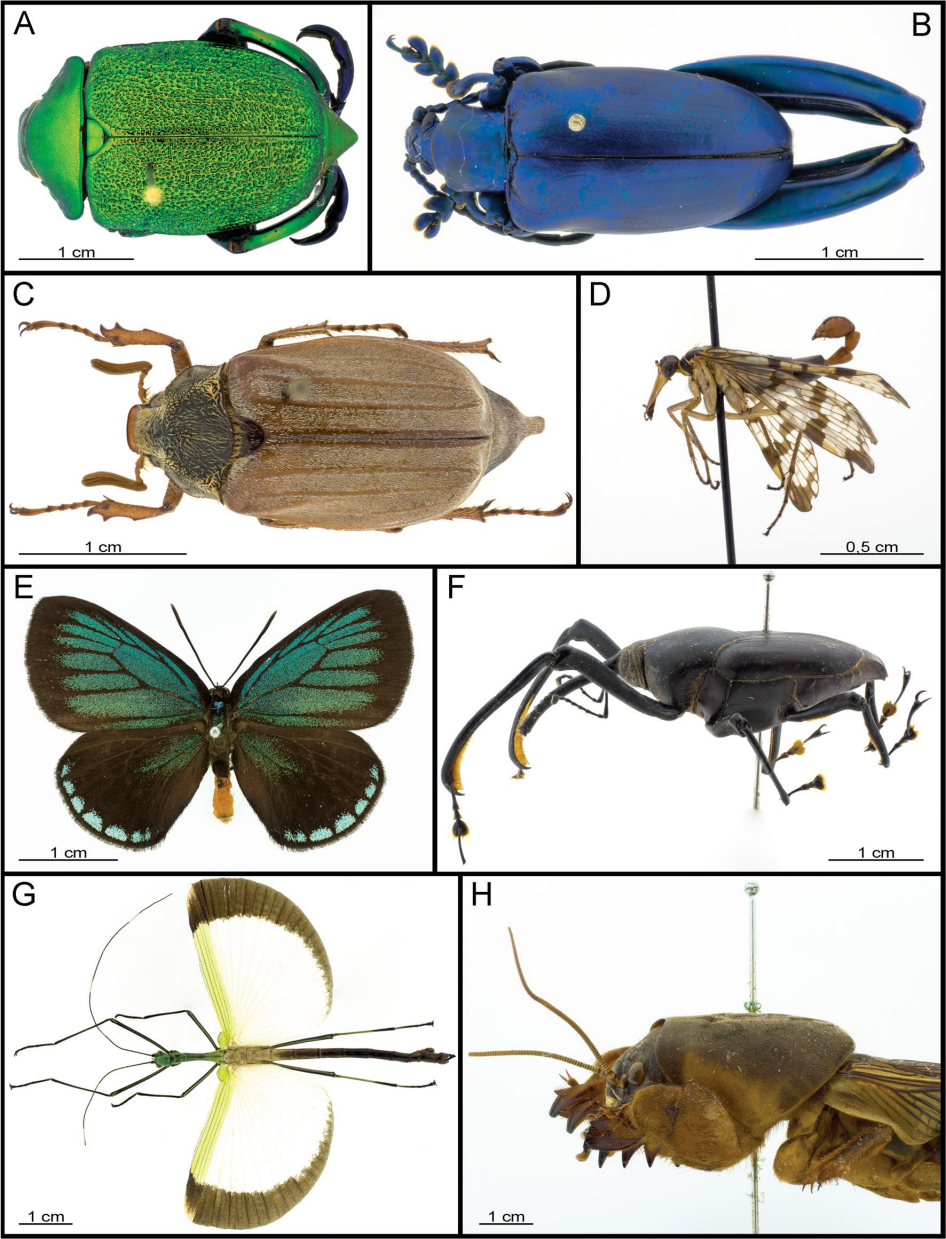
Insect specimens, left is anterior, except for E (up is anterior) : (**A**) *Chrysophora chrysochlora* (Coleoptera: Scarabaeidae), dorsal view; (**B**) *Sagra longicollis* (Coleoptera: Chrysomelidae), dorsal view; (**C**) *Melolontha melolontha* (Coleoptera: Scarabaeidae), dorsal view; (**D**) *Pannorpa communis* (Mecoptera: Panorpidae), lateral view; (**E**) *Eumaeus atala* (Lepidoptera: Lycaenidae), dorsal view; (**F**) *Macrochirus vittatus* (Coleoptera: Dryophthoridae), lateral view; (**G**) *Eurynecrosia nigrofasciata* (Phasmatodea: Lonchodidae), dorsal view; (**H**) *Gryllotalpa gryllotalpa* (Orthoptera: Gryllotalpidae), lateral view.

**Figure 5:**
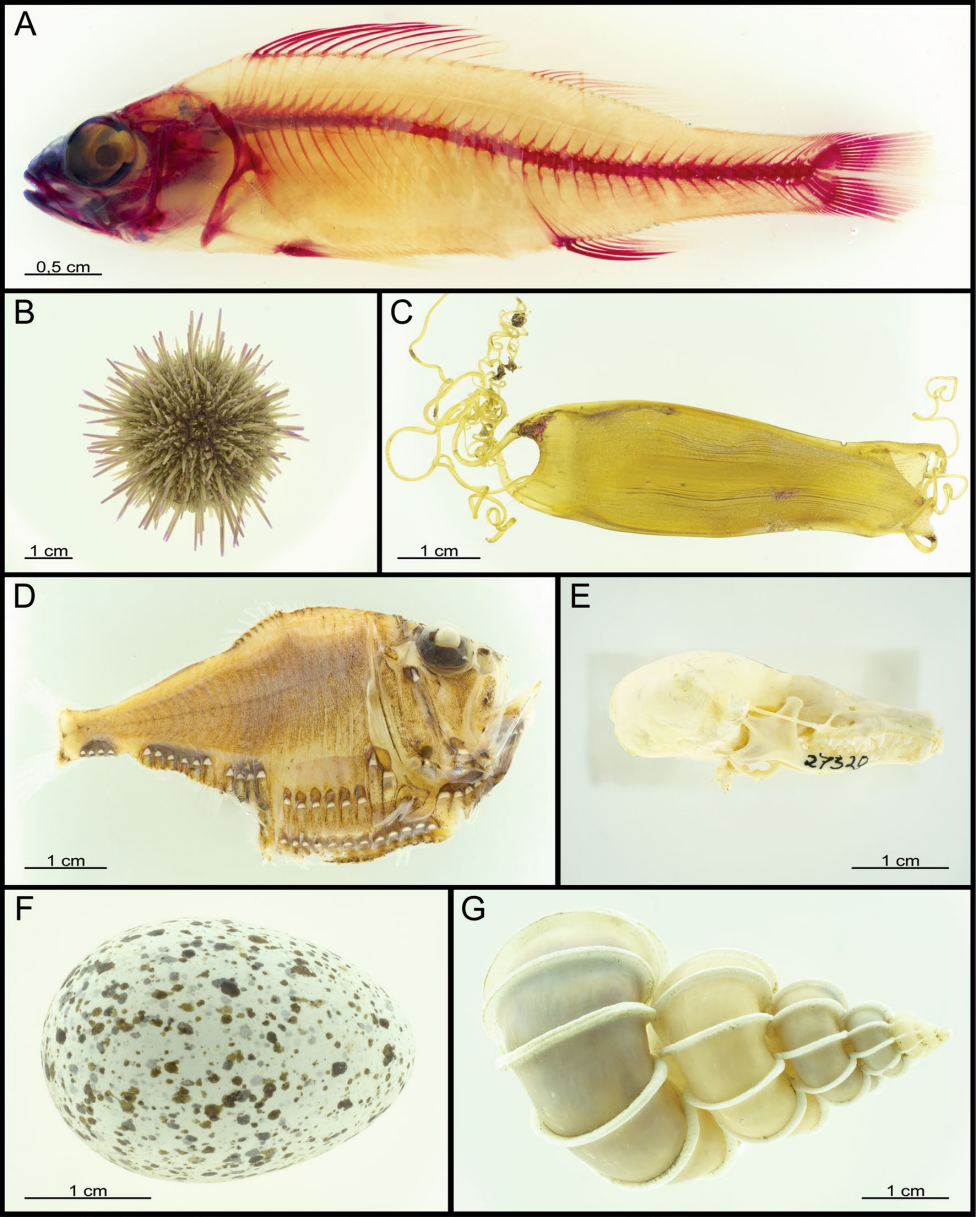
Other specimens: (**A**) *Perca fluviatilis* (Perciformes: Percidae), lateral view, left anterior; (**B**) *Psammechinus miliaris* (Camarodonta: Echinidae), aboral view; (**C**) *Scyliorhinus canicula* (Carcharhiniformes: Scyliorhinidae) egg; (**D**) *Argyropelecus* spec. (Stomiiformes: Stemoptychidae), lateral view, left anterior; (**E**) *Talpa europaea* (Eulipotyphla: Talpidae), lateral view, left anterior; (**F**) Jackdaw egg (Passeriformes: Corvidae); (**G**) *Epitonium* (Gastropoda: Epitoniidae) spec.

**Figure 6:**
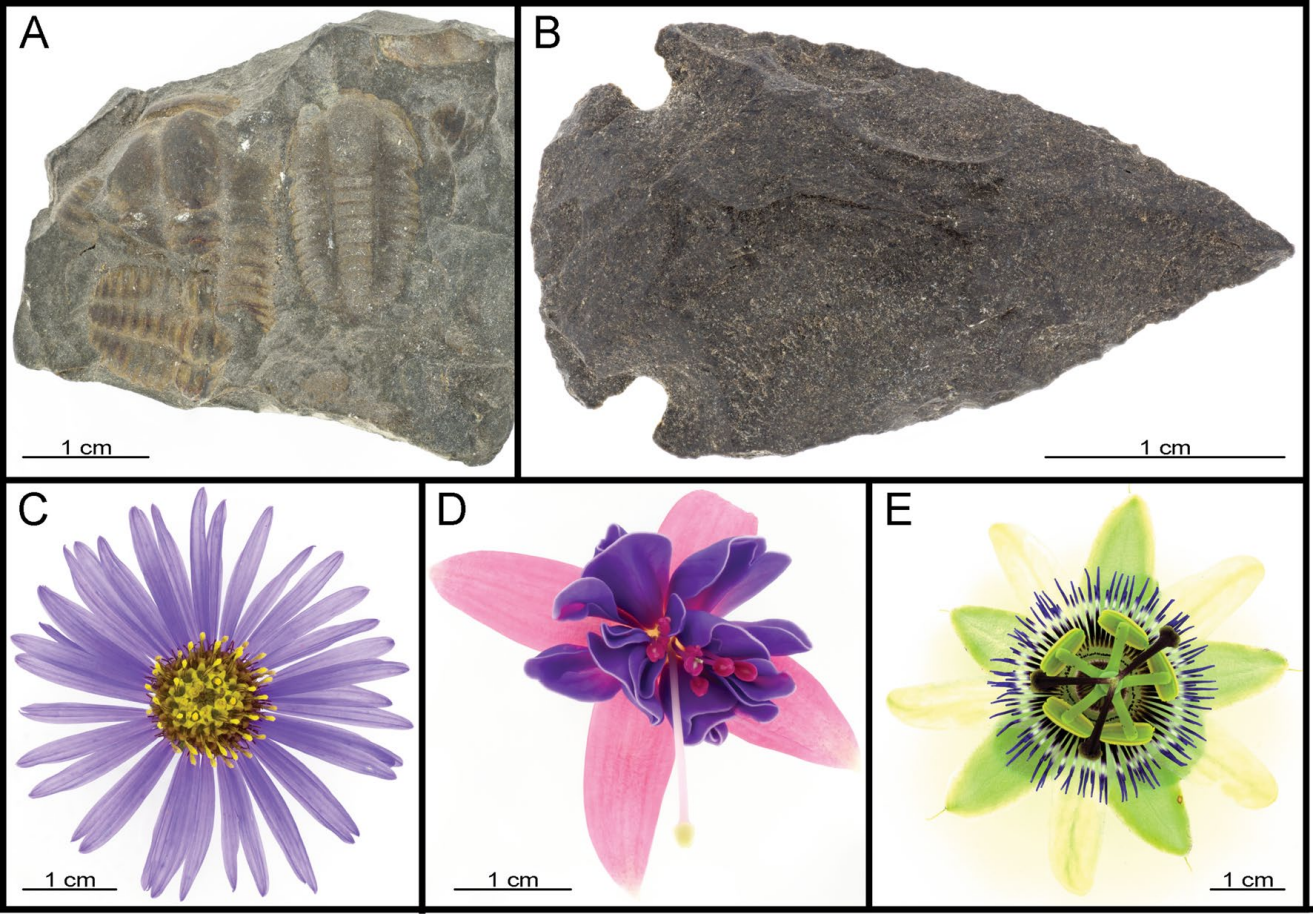
Objects and flowers: (**A**) *Ellipsocephalus hoffi* (Ptychopariida: Ellipsocephalidae); (**B**) Replica of the tip of an arrow; (**C**) *Aster alpinus* (Asterales: Asteraceae); (**D**) *Fuchsia* spec. (Myrtales: Onagraceae); (**E**) *Passiflora* spec. (Malpighiales: Passifloraceae).

Focus-stacked images of the same specimen were taken in a large sized dome (Fig. 1A,ld) with either warm or neutral white LED strips for comparison. Additional images showing the performance of our 3D-printed dome illumination system, using the small, medium and large sized dome with neutral white LED’s, are displayed in Figs. 4, 5 and 6. For larger objects, an extension plate, as well as a fitting light shielding ring, were placed on top of the dome table (Supplementary Information 3).

## Discussion

Commercial illumination solutions already exist—but they only fit on the equipment they were designed for and are expensive^20^. Kawada and Buffington^24^ already introduced a low-cost, self-made paper construction of an illumination dome. Although their dome produces impressive and high-quality results, like comparable commercial solutions (cf.^24^) or our system (cf. Figs. 4, 5, 6), the construction process is time consuming and requires a certain degree of dexterity. Beyond that, operating the system is more complicated, due to the fragility of the paper construction as well as the absence of a baseplate.

**Figure 7:**
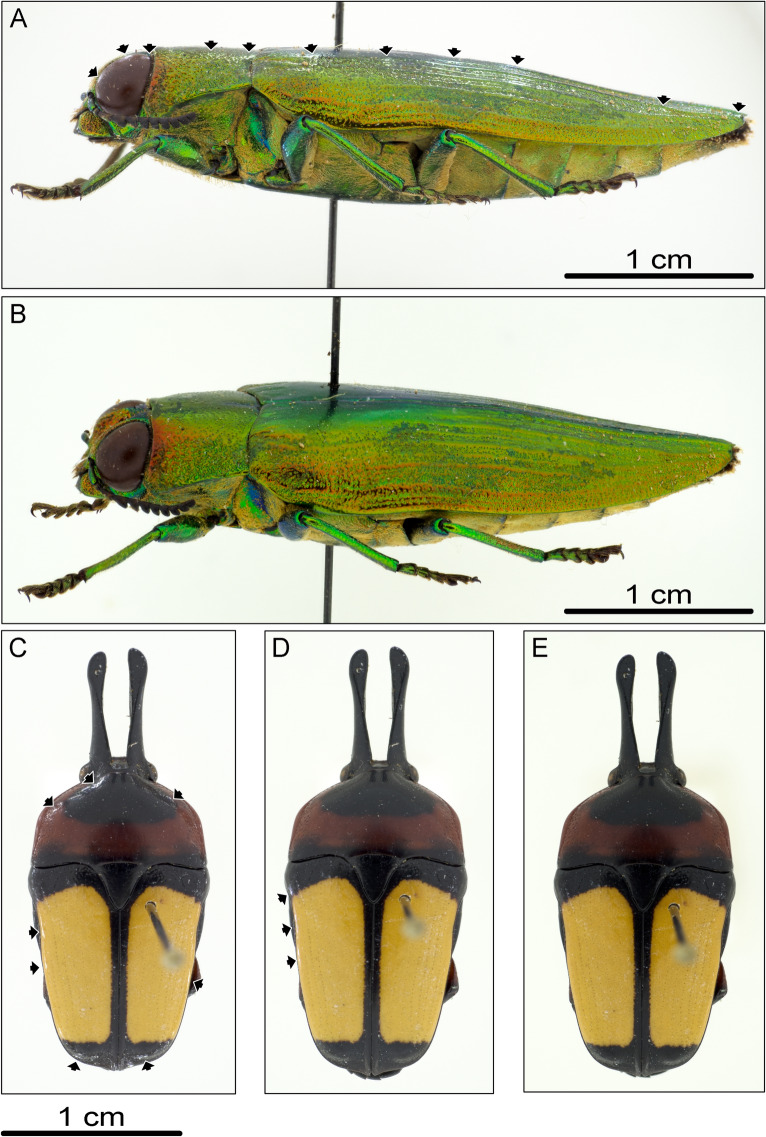
Consequences of light shielding ring, black arrows indicate reflections, left is anterior for (**A**,**B**), up is anterior for (**C**–**E**): (**A**) lateral view of the beetle Burprestidae (Coleoptera: Buprestidae), photographed without usage of light-shielding ring; (**B**) lateral view of the same specimen as in A, photographed using light-shielding ring; (**C**) dorsal view of the beetle *Dicheros bicornis* (Coleoptera: Scarabaeidae), photographed using 1 cm light-shielding ring; (**D**) dorsal view of *D. bicornis*, photographed using 2 cm light-shielding ring; (**E**) dorsal view of *D. bicornis*, photographed using 3 cm light-shielding ring.

**Figure 8:**
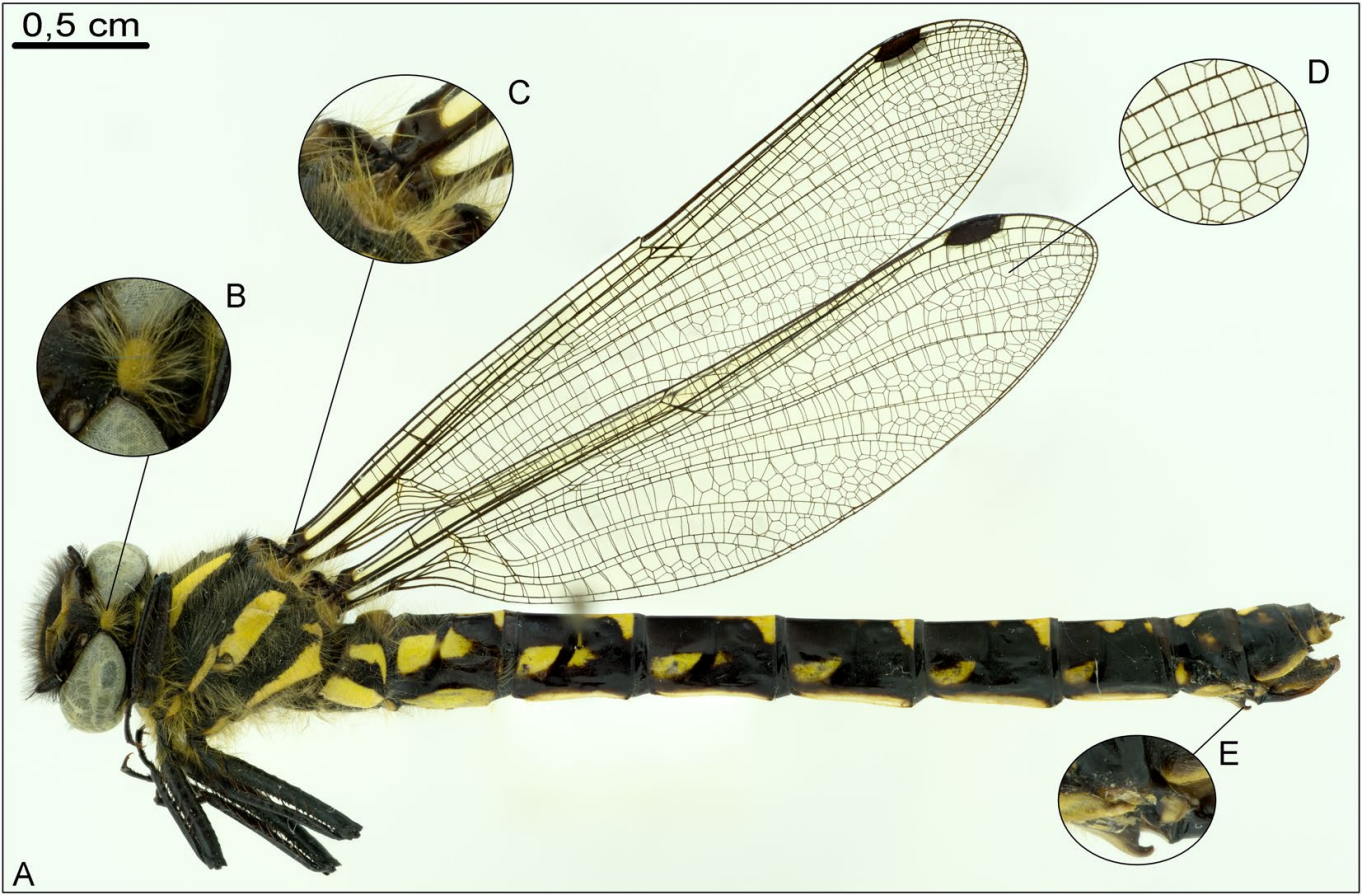
Dry specimen of the dragonfly *Epiophlebia susperstes* (Odonata, Epiophlebiidae), lateral view, left is anterior, stitched and stacked. (**A**) habitus overview; (further magnifications: **B**) dorsal head; (**C**) wing bases; (**D**) wing veins; (**E**) primary mating organ.

We built up on the idea of a self-made system, by creating a much sturdier 3D printed version of a hemispherical LED-illumination dome, strongly enhancing durability, leading to less assembling and next to no soldering effort required. Furthermore, the available sliding table allows for re-adjustments of the investigated object while inside the dome without any trouble (Fig. 1C,st). Moreover, the circular surface of the sliding table can be equipped with differently coloured 3D-printable inlays, to quickly adjust the background (Fig. 1C,iw,ib). Adding an iris diaphragm allows to adjust the upper opening of the dome (Fig. 3G). Kerr et al.^19^ suggested, to use an as small as possible upper opening, to avoid artefacts especially on iridescent objects. The iris diaphragm allows for an adjustment of the opening to the used lens and sample size. As our system relies on diffuse illumination, the light-shielding ring (Fig. 1C,lr) is a key component. As can be seen in Fig. 7A,B, using the light-shielding-ring protects the object from direct light of the LEDs and therefore, prevents reflections (as indicated by black arrows in Fig. 7A without ring, Fig. 7B with ring); the height of the ring may be adjusted, until all reflections are removed. Figure 7C–E shows how different light-shielding ring heights change the appearance of reflections (indicated by black arrows). Our findings support a ring with 3 cm height, but this may need to be enlarged depending on the size of the investigated object and its mounting height. Interestingly, by using only diffuse light, it is even possible to achieve meaningful images of objects in fluids, without any surface reflections (Fig. 5A,D) Furthermore, if working with larger objects (5 cm and up), it is necessary to scale up the dome or use an extension plate on top of the dome table as well as an appropriate sized light-shielding ring (Supplementary Information 3). Although the extension plate covers some LEDs, the remaining illumination strength is still sufficient (cf. Fig. 8). We recommend covering the bottom surface of the extension plate with light-tight material (e.g. black cardboard), to keep the LED light from shining through the extension plate.

It is recommended to print all parts using a FDM 3D-printer (also known as fused filament fabrication printer, FFF), where a strand of thermoplastic filament is melted and extruded through a small nozzle. The nozzle of this printer is movable along three axes, following a preprogramed path, to build the object. If a printer is available in the lab, the provided files can be prepared in a slicing program (e.g. Slic3r PE, Prusa Research s.r.o., Prague, Czech Republic), to convert the geometry into a file readable by the printer. As mentioned before, the printing settings we provide, may need further adjustments to your printer to get optimal results. Settings that most likely require adjustments, are printing bed temperature, extruder temperature and extrusion multiplier. While printing bed temperature is essential for proper print-to-bed adhesion, the extruder temperature is important for a smooth material flow from the nozzle and proper fusing of the printed layers. Furthermore, the value of the extrusion multiplier determines the amount of material that is deposited during the printing process.

As filament density tends to vary between different manufacturers, adjusting this value ensures a proper material flow. While too high values result in over extrusion, causing artefacts (e.g. unwanted plastic accumulations), too low values result in under extrusion, leading to strings of material that do not fuse properly, resulting in fragile surfaces. Additionally, apart from choosing the appropriate printing material, in terms of mechanical properties (e.g. durability, flexibility and heat resistance) and the maximum operating temperature of the thermoplastic material, one should also consider which layer thickness to use. It determines the resolution of the print along the z-axis: higher layer thickness leads to less resolution but faster prints, while a lower layer thickness leads to a finer surface, at the cost of longer printing times—so one must compromise for the best combination of these factors. If no 3D-printer is at hand, there are many local or online 3D-printing services, that can be commissioned, to print our provided models from a wide range of materials and in customized sizes.

For illumination, we decided to use a single LED ring for the small sized dome and a flexible LED strip for the medium and large sized dome. This minimizes soldering efforts, simplifying the system and speeding up the assembly process. In general, it requires the least assembling efforts if a flexible LED strip is available, including all connectors and power supply unit (no soldering needed). Unfortunately, most of the available systems in hardware stores (or similar) tend to have warm light LEDs. Results are equal to neutral light LEDs in terms of illumination power, yet the white balance has to be adjusted in image post processing. Nonetheless, the effort to assemble the neutral light version is worthy, as the authenticity of colour is much higher and it minimizes the magnitude of image post processing. In any case, the LED lights can be easily substituted if they are broken or if a better illumination source is found. On top of that, using a 12 V battery box (Fig. 1B) to power the dome, increases the mobility of the system, giving you the opportunity to use it in the field.

Of course, generating high quality images in general is dependent on the camera system in use. Although image resolution can be improved by focus-layer stacking and stitching (as shown in Fig. 2), the available camera equipment still has an important influence on image quality. Stacking and stitching can increase the level of detail drastically, but data size as well. The stacking and stitching process can be automated using up-to-date photo editing software, with normally very few to no manual adjustments are necessary. Generating those highly detailed images could be used to perform taxonomic or morphological studies and might improve the specimen digitizing process in museums (Fig. 8). A variety of different specimens and objects, featuring insects, molluscs, vertebrates, flowers and fossils—showing the comprehensive applications of our system—can be seen in Figs. 4, 5 and 6 and in Supplementary Information 6. Although, a grey background can promote visibility when it comes to fine details on the verge between object and background^21^; photography is still mingled with personal preferences, we used a neutral white background especially for colour recognition.

## Conclusions

Although commercial illumination solutions already exist—they only fit on the equipment they were designed for and tend to be expensive, making them less accessible. Kawada et al.^24^ already introduced a low-cost, self-made paper construction of a dome illumination system. Building up on that idea of a self-made system, we designed a much sturdier 3D-printed version of a hemispherical LED-illumination dome, with less assembling and next to no soldering effort required. Our system is (A) much more affordable than commercial solutions and (B) both modular and freely scalable, allowing it to be customized to the requirements of a particular setup. However, it is not just an improvement in terms of endurance, but also includes new optional features like a sliding table to arrange the investigated object while inside the dome (especially helpful for small animals at high magnification) and an iris diaphragm, to maximize light yield. Overall, the 3D-printed dome represents an improvement to the paper dome by Kawada and Buffington^24^, truly being able to compete with commercial solutions. As such, our dome represents an important improvement of scientific illumination methods, highly increasing its accessibility to the scientific community, hopefully proving to be a useful tool for many studies in the future. Because of its low costs and easy reproducibility, especially students, labs and in general, people on a low budget now have the possibility to generate meaningful and professional images to finally compete with cutting edge labs. We here present everything you need to set up your own LED dome illumination system, designed for easy assemblage, durability and flexibility. Allowing every user to freely adjust the dome system to their own needs and this with minimized effort, costs and construction time.

## Supplementary information

Supplementary information 1

Supplementary information 2

Supplementary information 3

Supplementary information 4

Supplementary information 5

Supplementary information 6

## Data Availability

All '.stl' files are available to download on https://www.thingiverse.com/FabsenMcForester/designs.

## References

[1] Michels J, Vogt J, Simon P, Gorb SN (2015). New insights into the complex architecture of siliceous copepod teeth. Zoology.

[2] Matsumura Y, Kovalev AE, Gorb SN (2017). Penetration mechanics of a beetle intromittent organ with bending stiffness gradient and a soft tip. Sci. Adv..

[3] Büsse S, Gorb SN (2018). Material composition of the mouthpart cuticle in a damselfly larva (Insecta: Odonata) and its biomechanical significance. R. Soc. Open Sci..

[4] Bäumler F, Büsse S (2019). Resilin in the flight apparatus of Odonata (Insecta)—cap tendons and their biomechanical importance for flight. Biol. Lett..

[5] Hörnschemeyer T, Beutel RG, Pasop F (2002). Head Structures of *Priacma serrata* Leconte (Coleptera, Archostemata) inferred from X-ray tomography. J. Morphol..

[6] Wipfler B, Klug R, Ge S, Bai M, Göbbels J, Yang X, Hörnschemeyer T (2014). The thorax of Mantophasmatodea, the morphology of flightlessness, and the evolution of the neopteran insects. Cladistics.

[7] Büsse S, Hörnschemeyer T, Gorb SN (2017). The head morphology of *Pyrrhosoma nymphula* larvae (Odonata: Zygoptera) focusing on functional aspects of the mouthparts. Front. Zool..

[8] Bäumler F, Gorb SN, Büsse S (2018). Comparative morphology of the thorax musculature of adult Anisoptera (Insecta: Odonata): Functional aspects of the flight apparatus. Arthropod Struct. Dev..

[9] Friedrich F, Beutel RG (2008). Micro-computer tomography and a renaissance of insect morphology. Proc. SPIE Dev. X-Ray Tomogr..

[10] Steinhoff PO, Uhl G (2015). Taxonomy and nomenclature of some mainland SE-Asian *Coeliccia* species (Odonata, Platycnemididae) using micro-CT analysis. Zootaxa.

[11] Büsse S (2016). Morphological re-examination of *Epiophlebia laidlawi* (Insecta: Odonata) including remarks on taxonomy. Int. J. Odonatol..

[12] Beutel RG, Zimmermann D, Krauß M, Randolf S, Wipfler B (2010). Head morphology of *Osmylus fulvicephalus* (Osmylidae, Neuroptera) and its phylogenetic implications. Organ. Divers. Evol..

[13] Beutel RG, Yan E, Richter A, Büsse S, Miller KB, Yavorskaya M, Wipfler B (2017). The head of *Heterogyrus milloti* (Coleoptera: Gyrinidae) and its phylogenetic implications. Arthropods Syst. Phylogeny.

[14] Beutel RG, Yan E, Yavorskaya M, Büsse S, Gorb SN, Wipfler B (2019). On the thoracic anatomy of the Madagascan *Heterogyrus milloti* and the phylogeny of Gyrinidae (Coleoptera). Syst. Entomol..

[15] Büsse S, Heckmann S, Hörnschemeyer T, Bybee SM (2018). The phylogenetic relevance of thoracic musculature: A case study including a description of the thorax anatomy of Zygoptera (Insecta: Odonata) larvae. Syst. Entomol..

[16] Büsse S, Büscher TH, Heepe L, Gorb SN (2019). Adaptations of dragonfly larvae and their exuviae (Insecta: Odonata), attachment devices and their crucial role during emergence. J. Insect Physiol..

[17] Baird RC (2010). Leveraging the fullest potential of scientific collections through digitization. Biodivers. Inform..

[18] Ströbel B, Schmelzle S, Blüthgen N, Heethoff M (2018). An automated device for the digitization and 3D modelling of insects, combining extended-depth-of-field and all-side multi-view imaging. ZooKeys.

[19] Kerr PH, Fisher EM, Buffington ML (2008). Dome lighting for insect imaging under a microscope. Am. Entomol..

[20] Vollmar A, Macklin JA, Ford LS (2010). Natural history specimen digitization: Challenges and concerns. Biodivers. Inform..

[21] Buffington ML, Burks RA, McNeil LA (2005). Advanced techniques for imaging microhymenoptera. Am. Entomol..

[22] Buffington ML, Gates M (2008). Advanced imaging techniques II: Using a compound microscope for photographing point-mount specimens. Am. Entomol..

[23] Hunter F, Biver S, Fuqua P (2011). Light Science and Magic: An Introduction to Photographic Lighting.

[24] Kawada R, Buffington ML (2016). A scalable and modular dome illumination system for scientific microphotography on a budget. PLoS One.

[25] Dominugue MJ, Pulsifer DP, Lakhtakia A, Berkebile J, Steiner KC, Lelito JP, Hall LP, Barker TC (2015). Detecting emerald ash borers (*Agrilus planipennis*) using branch traps baited with 3D-printed beetle decoys. J. Pest. Sci..

[26] Berry D, Selby RD, Horvath JC, Cameron RH, Porqueras D, Stouthammer R (2016). A modular system of 3D printed emergence traps for studying the biology of shot hole borers and other Scolytinae. J. Econ. Entomol..

[27] Will KW, Steplowski I (2016). A 3D printed malaise trap head. Pan-Pac. Entomol..

[28] Horton DR, Miliczky ER, Lewis TM, Wohleb CH, Waters TD, Dickens AA, Halbert S, Ramadugu C, Jensen AS (2018). Building a better Psylloidea (Hemiptera) trap? A field-look at a prototype trap constructed using three-dimensional printer technology. Can. Entomol..

[29] Mendez PK, Lee S, Venter CE (2018). Imaging natural history museum collections from the bottom up: 3D print technology facilitates imaging of fluid-stored arthropods with flatbed scanners. ZooKeys.

[30] Byagathvalli G, Pomerantz A, Sinha S, Standeven J, Bhamla MS (2019). A 3D-printed hand-powered centrifuge for molecular biology. PLoS One.

[31] Hoshi (2019). Field testing of a lightweight, inexpensive, and customisable 3D-printed mosquito light trap in the UK. Sci. Rep..

