# Supplementary material for: Illuminating nature’s beauty: modular, scalable and low-cost LED dome illumination system using 3D-printing technology
Source: Sci Rep. 2020 Jul 22;10:12172. doi: 10.1038/s41598-020-69075-y (PMC7376240; doi:10.1038/s41598-020-69075-y)
Supplement: Supplementary file 1 — Supplementary information 1 [file 41598_2020_69075_MOESM1_ESM.pdf]

Supplementary information A

Illuminating nature's beauty - modular, scalable and low-cost LED dome illumination system using 3D-printing technology

Fabian Bäumler, Alexander Koehnsen, Halvor T. Tramsen, Stanislav N. Gorb and Sebastian Büsse

	Species	Place	Date	Size (cm)
1	*Amaurodes passerini*	Chingoma farm, Mrurwi, Mashonatand, Zimbabwe	Januar 04	2,8
2	*Argyropelecus*	Mid Atlantik	Juni 05	6,1
3	Aster alpinus	Botanischer Garten, Kiel	August 19	4,3
4	Bupristidae	Chiang Mai, Thailand	März 05	3,6
5	*Caiman crocodylus*	Zoo	Mai 05	9,2
6	*Carabus auratus*	Ratingen, Kr. Neuhaus	Juli 84	2,6
7	*Chrysochroa buquetii buquetii*	Cameon Highlands, Malaysia	Juni 03	4,2
8	*Chrysophora chrysochlora*	Satipo, Peru	März 03	2,9
9	*Cyclommatus metallifer finae*	Peleng, Indonesien	April 04	3,6
10	*Dares verrucosus verrucosus*	Borneo, Sabah, Spilok near Sandakan	August 84	3,4
11	*Dicheros bicornis*	Cameron Highlands, Malaysia	August 02	1,6
12	*Ellipsocephalus hoffi*	Jince, Tschechien	Kambrium, ca. 500 millionen Jahre	4
13	*Epiophlebia superstes*	-	-	4,9
14	*Epitonium spec.*	-	-	5,5
15	*Eumacus atala*	Miami, Florida	-	4
16	*Eurynecroscia nigrofasciata*	Tapah Hills, Perak, Malaysia	März 13	5,8
17	Fuchsia spec.	Botanischer Garten, Kiel	August 19	3,5
18	*Golofa claviger*	Carpich, Huanuco, Peru	Januar 04	3,8
19	*Gryllotalpa gryllotalpa*	Freiburg, Deutschland	Mai 25	6,3
20	*Jackdaw egg*	-	-	3
21	*Lasiocampa trifolii*	Sachsen, Zitzschewig	-	4,2
22	*Lamprima adolphinae*	Mt. Arfak W. Irian, Indonesia	Mai 04	3
23	*Lemmus sibiricus*	Bikada, Taimyr-Halbinsel, Russland	September 91	3,2
24	*Macrochirus vittatus*	Borneo	-	3,2
25	*Melolontha melolontha*	Heiterau, Feldweg	Mai 77	2,5
26	*Pannorpa communis*	Rosensee	Juli 17	1,1
27	*Passiflora spec.*	Botanischer Garten, Kiel	August 19	5,4
28	*Perca fluviacilis*	-	Juli 05	6
29	*Philanthus triangulum*	Rosensee	Juli 17	1

30	*Phyllium pulchrifolium*	Sukabumi W-Java	September 16	5,8
31	*Psammechinus miliaris*	-	-	3,5
32	Replikat einer Pfeilspitze	-	-	3,2
33	*Sagra longicollis*	Wong Chin, Phare, Thailand	Juni 04	2,3
34	*Scyliorhinus canicula*	Geomar Aquarium Kiel	Juni 18	5,5
35	*Talpa europaea*	Travenhorst, Krs. Segeberg	Juli 87	3,5
