# Supplementary material for: Illuminating nature’s beauty: modular, scalable and low-cost LED dome illumination system using 3D-printing technology
Source: Sci Rep. 2020 Jul 22;10:12172. doi: 10.1038/s41598-020-69075-y (PMC7376240; doi:10.1038/s41598-020-69075-y)
Supplement: Supplementary file 2 — Supplementary information 2 [file 41598_2020_69075_MOESM2_ESM.pdf]

Supplementary information B

Illuminating nature's beauty - modular, scalable and low-cost LED dome illumination system using 3D-printing technology

Fabian Bäumler, Alexander Koehnsen, Halvor T. Tramsen, Stanislav N. Gorb and Sebastian Büsse

Setting	Value
Plater	
Print settings	0.20mm QUALITY MK3
Generic PLA	Generic PLA
Supports	None
Infill	15%
Layers and Perimeters	
Layer height	0.2 mm
First layer height	0.2 mm
Perimeters	2
Solid layers	Top: 5; Bottom: 4
Ensure vertical shell thickness	yes
Seam position	nearest
Skirt and Brim	
Loops minimum	1
Distance from object	2 mm
Skirt height	3 layers
Minimal filament extrusion length	4 mm
Brim width	0 mm
Speed for print moves	
Perimeters	45 mm/s
Small perimeters	25 mm/s
External perimeters	25 mm/s
Infill	80 mm/s
Solid infill	80 mm/s
Top solid infill	40 mm/s
Support material	50 mm/s
Bridges	30 mm/s
Gap fill	40 mm/s

Travel for non-print moves	180 mm/s
First layer speed	20 mm/s
Abdvanced Extrusion width, Flow and Slicing	
Default extrusion width	0.45 mm
First layer	0.42 mm
Perimeters	0.45 mm
External perimeters	0.45 mm
Infill	0.45 mm
Solid infill	0.45 mm
Top solid infill	0.45 mm
Support material	0.35 mm
Bridge flow ratio	0.95
Slice gap closing radius	0.049 mm
Elephant foot compensation	0
Filament Settings	
Extrusion multiplier	1
Extruder temperature in °C	215
Bed temperature in °C	60
Keep fan always on and enable auto cooling	Yes
Printer Settings	
Nozzle diameter	0.4 mm
Layer height from minimum to maximum	0.07 mm – 0.25 mm
Retraction	
Length	0.8 mm
Lift Z	0.6 mm
Only lift Z	Above Z: 0 mm; Below Z: 209 mm
Retraction Speed	35 mm/s
Deretraction Speed	0 mm/s
Extra length on restart	0 mm/s

Minimum travel after retraction	1 mm
Retract on layer change	Yes
Wipe while retracting	Yes
Retract amount before wipe	0 %
