# Supplementary material for: Illuminating nature’s beauty: modular, scalable and low-cost LED dome illumination system using 3D-printing technology
Source: Sci Rep. 2020 Jul 22;10:12172. doi: 10.1038/s41598-020-69075-y (PMC7376240; doi:10.1038/s41598-020-69075-y)
Supplement: Supplementary file 3 — Supplementary information 3 [file 41598_2020_69075_MOESM3_ESM.pdf]

Supplementary information C

Illuminating nature’s beauty - modular, scalable and low-cost LED dome illumination system using 3D-printing technology

Fabian Bäumler, Alexander Koehnsen, Halvor T. Tramsen, Stanislav N. Gorb and Sebastian Büsse

Print for:	Small Dome		Medium Dome		Large Dome	
Dome						
	Plain	Iris	Plain	Iris	Plain	Iris
Sdome	1	-	-	-	-	-
Mdome	-	-	1	-	-	-
MdomeIris	-	-	-	1	-	-
Mring (1x)	-	-	-	1	-	-
Mtopring (1x)	-	-	-	1	-	-
Mspacer (4x)	-	-	-	4	-	-
Mblade (11x)	-	-	-	11	-	-
Mnubsi (11x)	-	-	-	11	-	-
Ldome	-	-	-	-	1	-
LdomeIris	-	-	-	-	-	1
Lring (1x)	-	-	-	-	-	1
Ltopring (1x)	-	-	-	-	-	1
Lspacer (4x)	-	-	-	-	-	4
Lblade (11x)	-	-	-	-	-	11
Lnubsi (11x)	-	-	-	-	-	11
Plate						
	Plain	Table	Plain	Table	Plain	Table
Splate	1	-	-	-	-	-
SplateT	-	1	-	-	-	-
Stable	-	1	-	-	-	-
Sbottom	-	1	-	-	-	-
Slightring	1	1	-	-	-	-
Mplate	-	-	1	-	-	-
MplateT	-	-	-	1	-	-
Mtable	-	-	-	1	-	-
Mbottom	-	-	-	1	-	-
Mlightring	-	-	1	1	-	-
Lplate	-	-	-	-	1	-
LplateT	-	-	-	-	-	1
Ltable	-	-	-	-	-	1
Lbottom	-	-	-	-	-	1
Llightring	-	-	-	-	1	1
Optional						
Extension plate				Explate		
Light-shielding ring				Exlightring		
Medium table with hole				Mtableh		

Large table with hole	LtableH
Inlay for large and medium table with hole	MLtableI
