# Supplementary material for: Illuminating nature’s beauty: modular, scalable and low-cost LED dome illumination system using 3D-printing technology
Source: Sci Rep. 2020 Jul 22;10:12172. doi: 10.1038/s41598-020-69075-y (PMC7376240; doi:10.1038/s41598-020-69075-y)
Supplement: Supplementary file 4 — Supplementary information 4 [file 41598_2020_69075_MOESM4_ESM.pdf]

Supplementary information D

Illuminating nature's beauty - modular, scalable and low-cost LED dome illumination system using 3D-printing technology

Fabian Bäumler, Alexander Koehnsen, Halvor T. Tramsen, Stanislav N. Gorb and Sebastian Büsse

Material	Approximate costs (Euro)	Source
PLA	21	https://shop.prusa3d.com/en/filament/39-white-pla-filament-1kg.html
LED (warm)	30	Hardware store https://www.bauhaus.info/kuechenleuchten-unterbauleuchten/paulmann-led-band-flexled-3d-basisset/p/26016535
LED (neutral)	11,9	https://www.reichelt.de/led-streifen-neutralweiss-6400-k-5000-mm-vt-2561-p253583.html?&trstct=pol_21
Screws	0,5	Hardware store
Power supply unit	12	https://www.amazon.de/gp/product/B071KXCRBM/ref=ppx_yo_dt_b_asin_image_o08_s00?ie=UTF8&psc=1
Connector with switch	9	https://www.amazon.de/gp/product/B01GQ3I1Y6/ref=ppx_yo_dt_b_asin_image_o08_s00?ie=UTF8&psc=1
LED ring	8,5	https://www.amazon.de/LED-Ring-SMD-50-mm/dp/B07ML3PTYS/ref=asc_df_B07MHHMDB6/?tag=&linkCode=df0&hvadid=309758879670&hvpos=1o2&hvnetw=g&hvrand=2807500279670552635&hvpone=&hvptwo=&hvqmt=&hvdev=c&hvdvcmdl=&hvlocint=&hvlocphy=9060698&hvtargid=pla-724047001394&th=1&ref=&adgrpid=61401833413
Battery box	8,5	https://www.amazon.de/gp/product/B078RD44XR/ref=ppx_yo_dt_b_asin_title_o00_s00?ie=UTF8&psc=1

Additional tools:
Hot glue gun
Screwdriver
Scissors
Soldering equippment
Glue
Hot glue
