# Supplementary material for: Illuminating nature’s beauty: modular, scalable and low-cost LED dome illumination system using 3D-printing technology
Source: Sci Rep. 2020 Jul 22;10:12172. doi: 10.1038/s41598-020-69075-y (PMC7376240; doi:10.1038/s41598-020-69075-y)
Supplement: Supplementary file 6 — Supplementary information 6 [file 41598_2020_69075_MOESM6_ESM.pdf]

Supplementary information E

Illuminating nature’s beauty - modular, scalable and low-cost LED dome illumination system using 3D-printing technology

Fabian Bäumler, Alexander Koehnsen, Halvor T. Tramsen, Stanislav N. Gorb and Sebastian Büsse

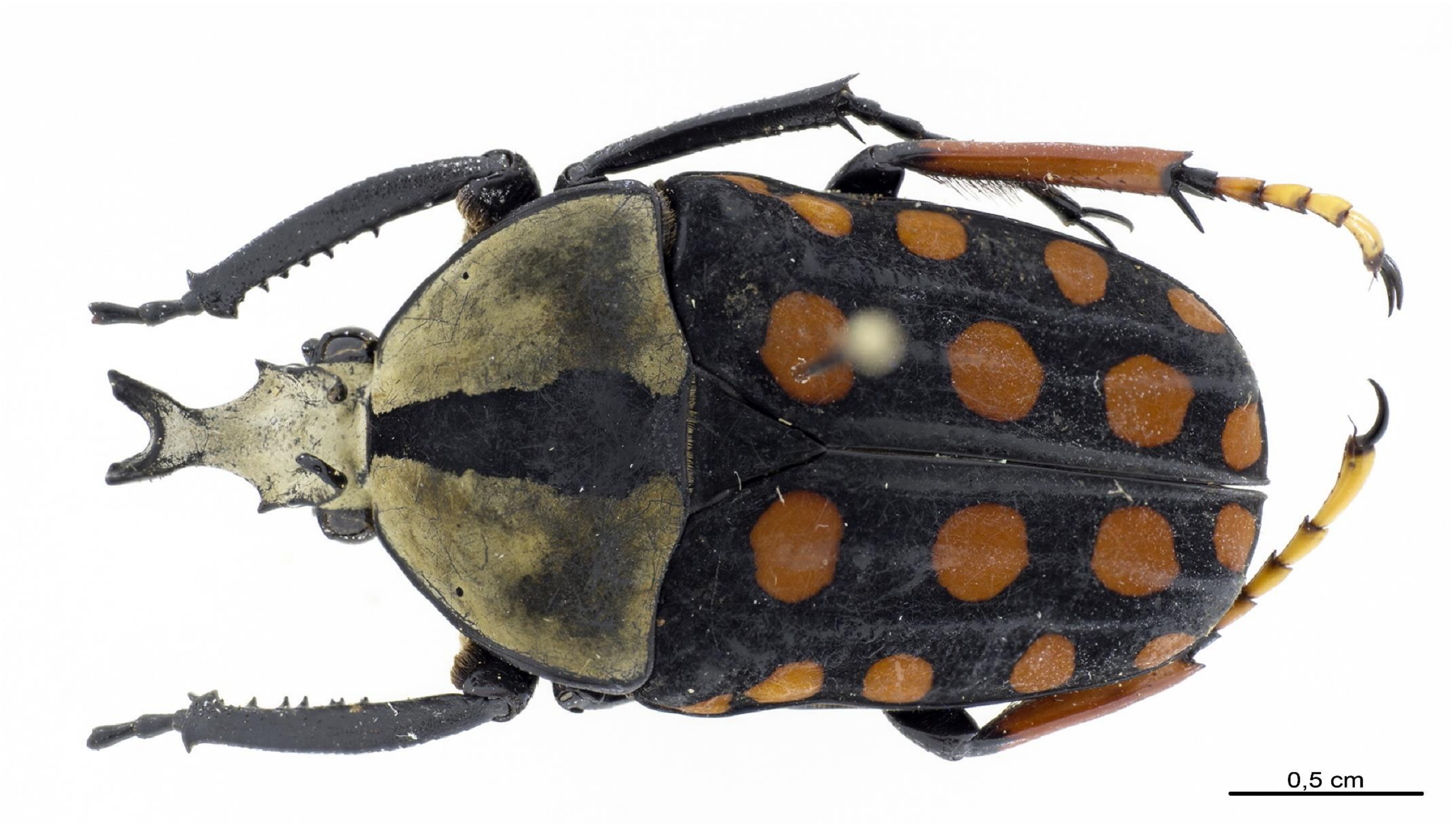

E.1 – *Amaurodes passerine*

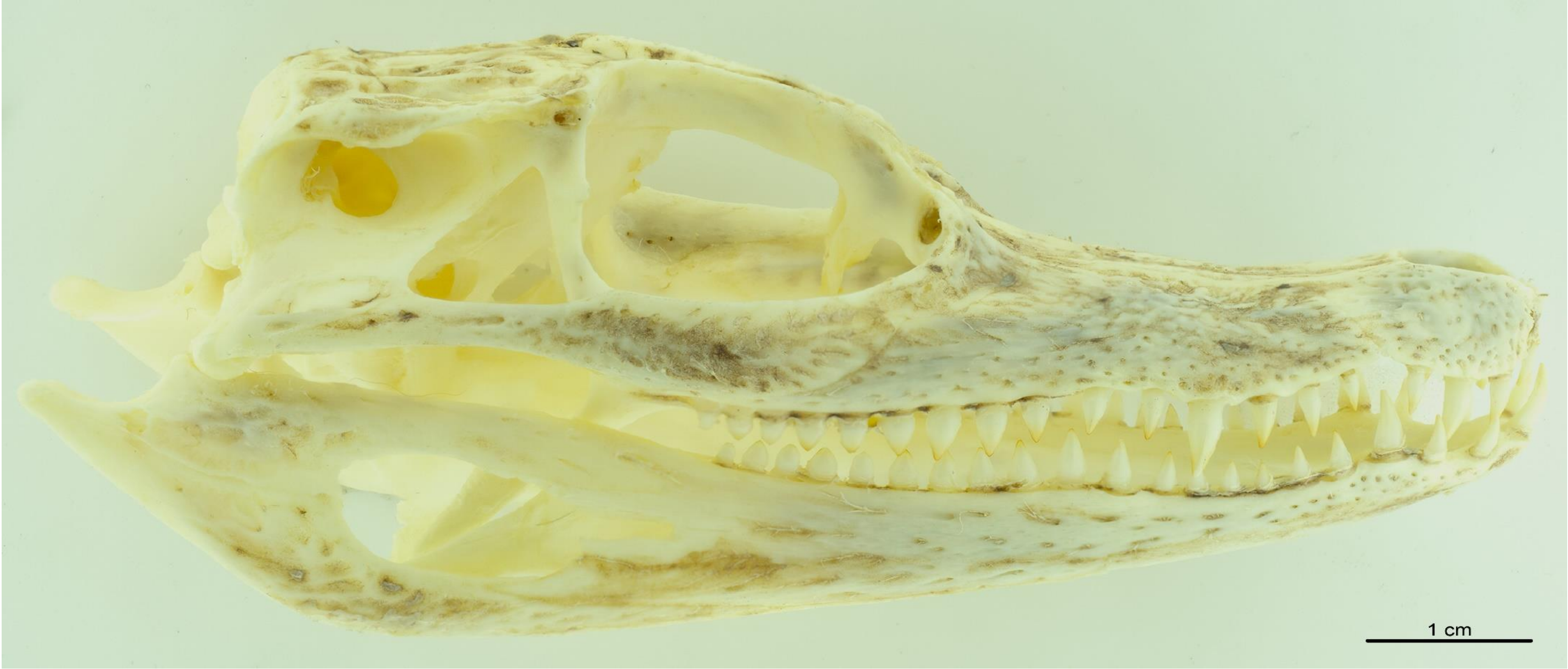

E.2 – *Caiman crocodilus*

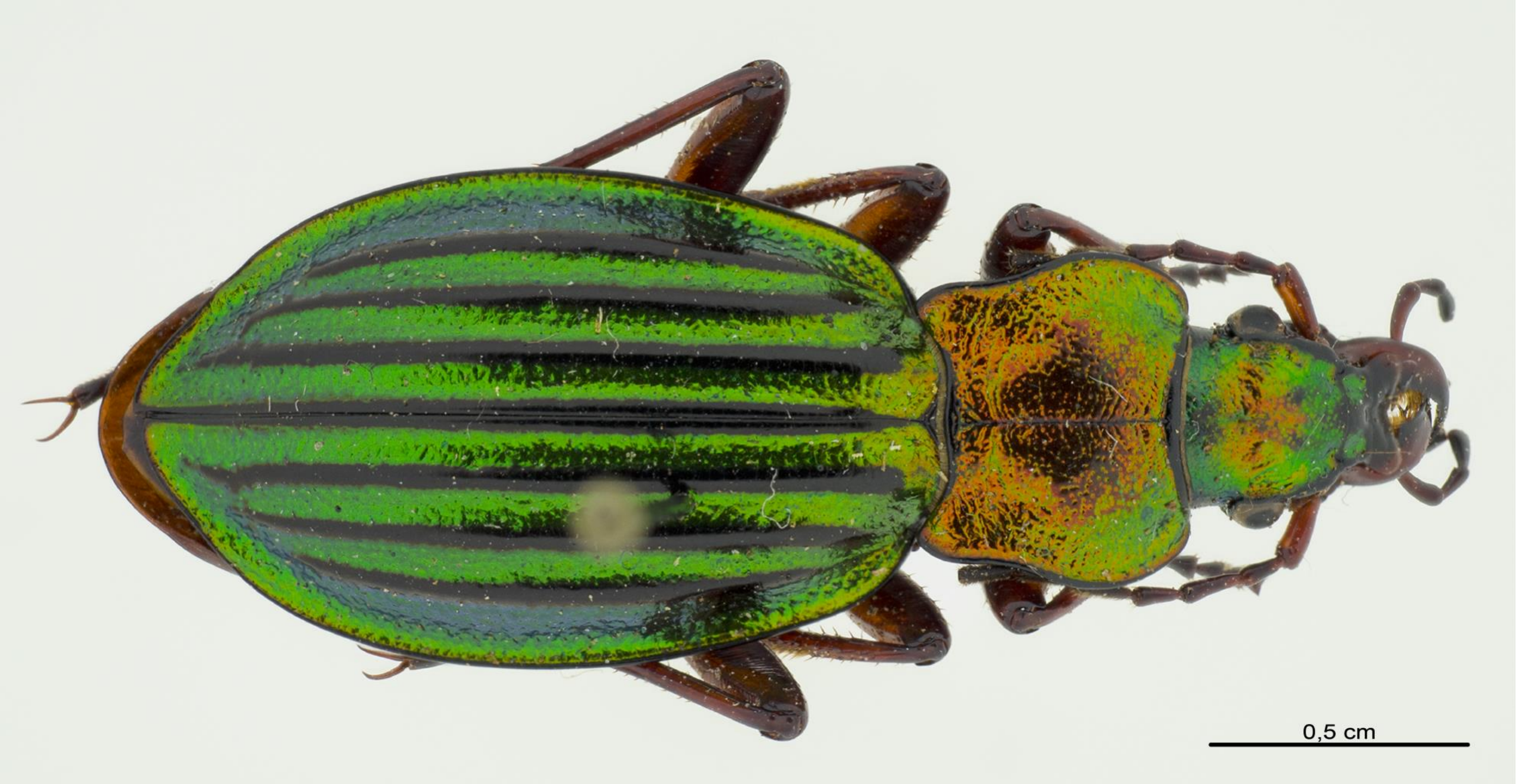

E.3 – *Carabus auratus*

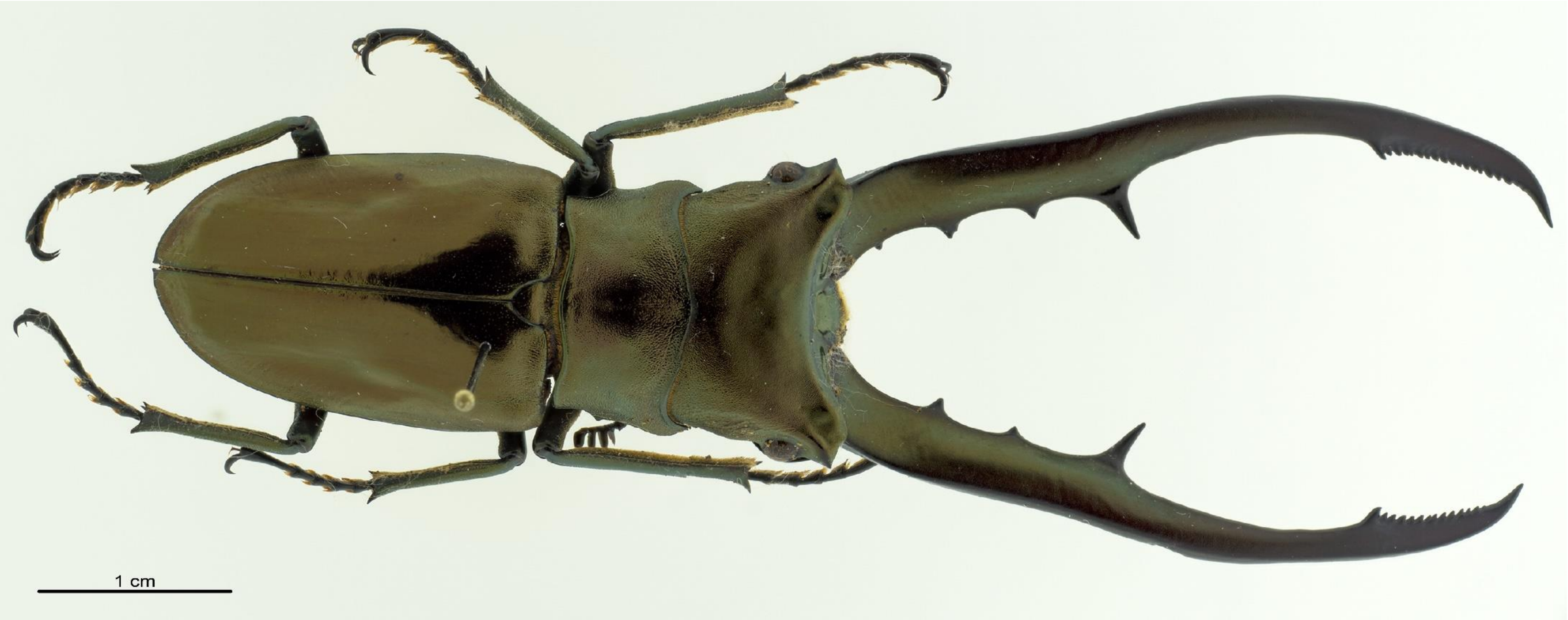

E.4 – *Cyclommatus metallifer*

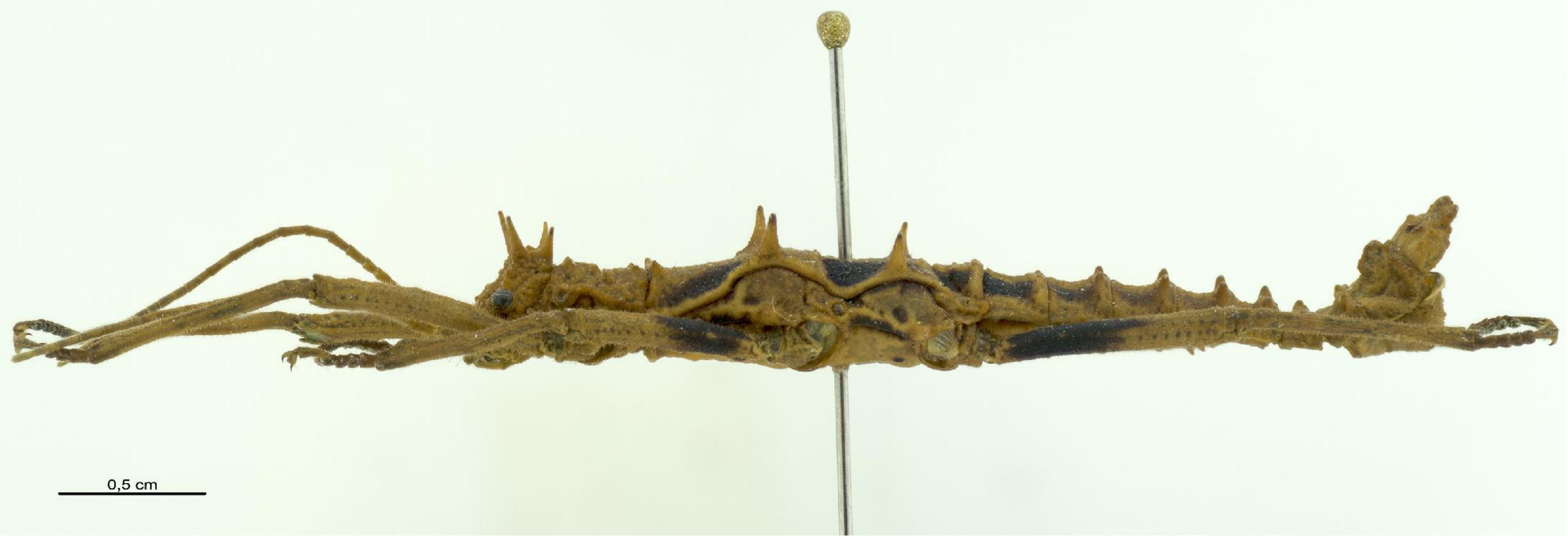

E.5 – *Dares verrucosus*

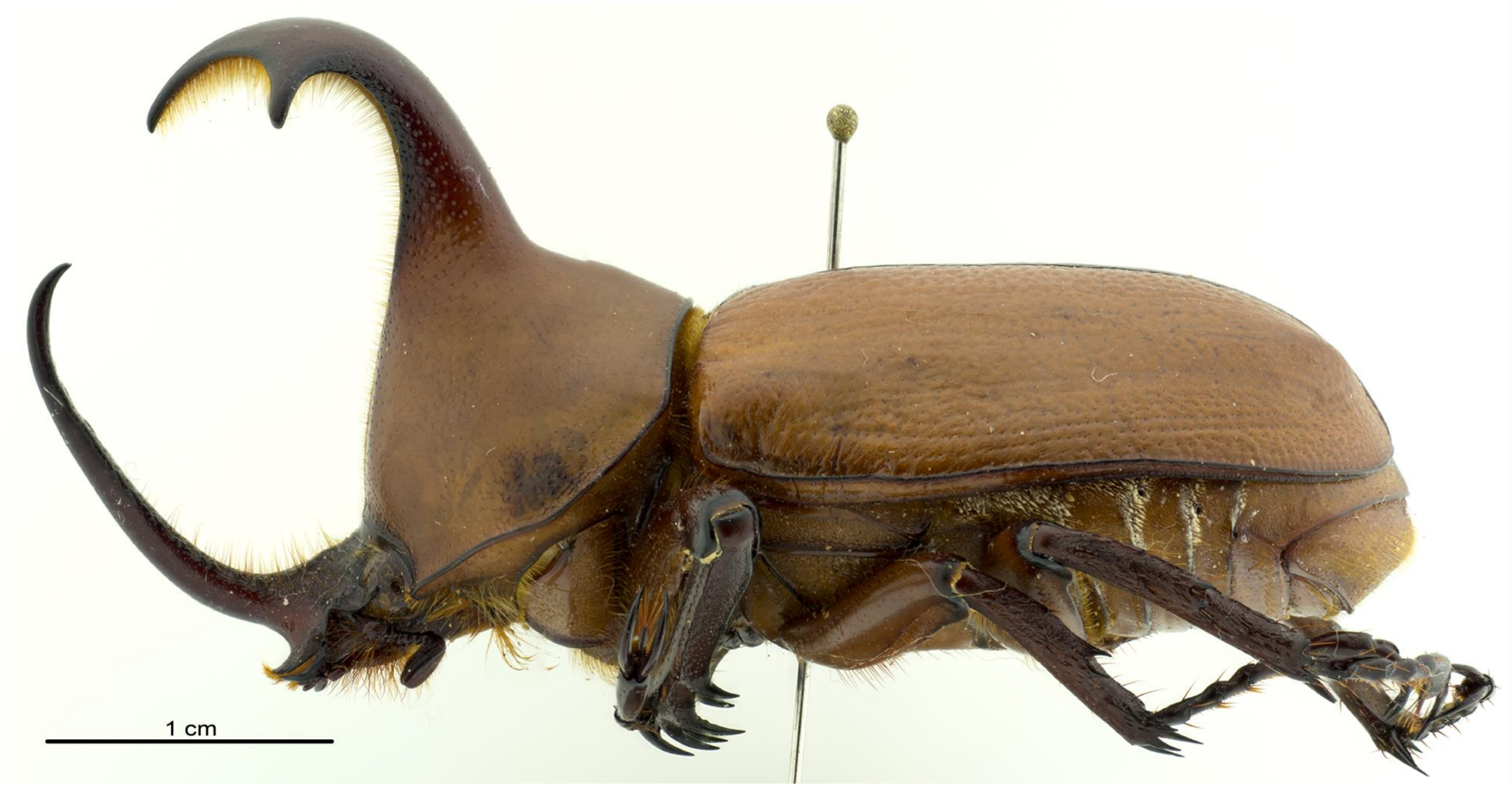

E.6 – *Golofer claviger*

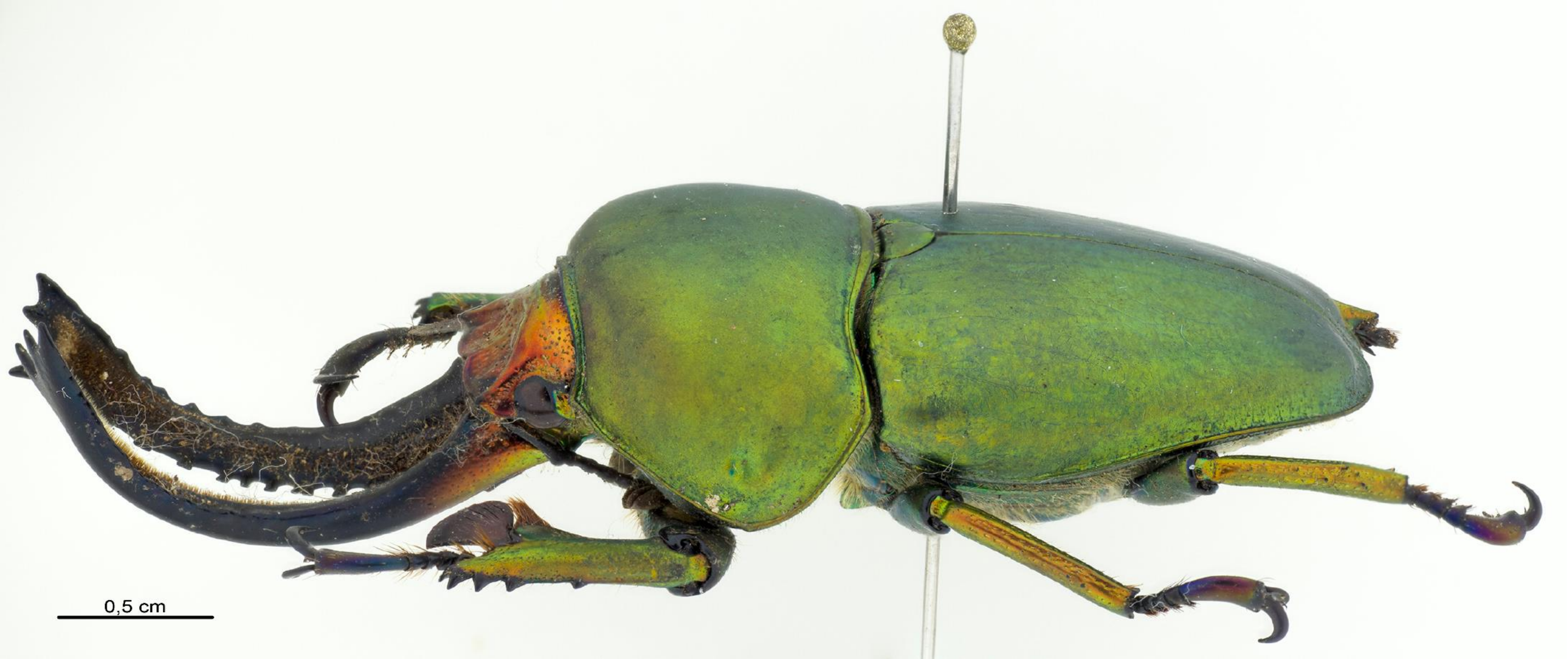

E.7 – *Laprima adolphinae*

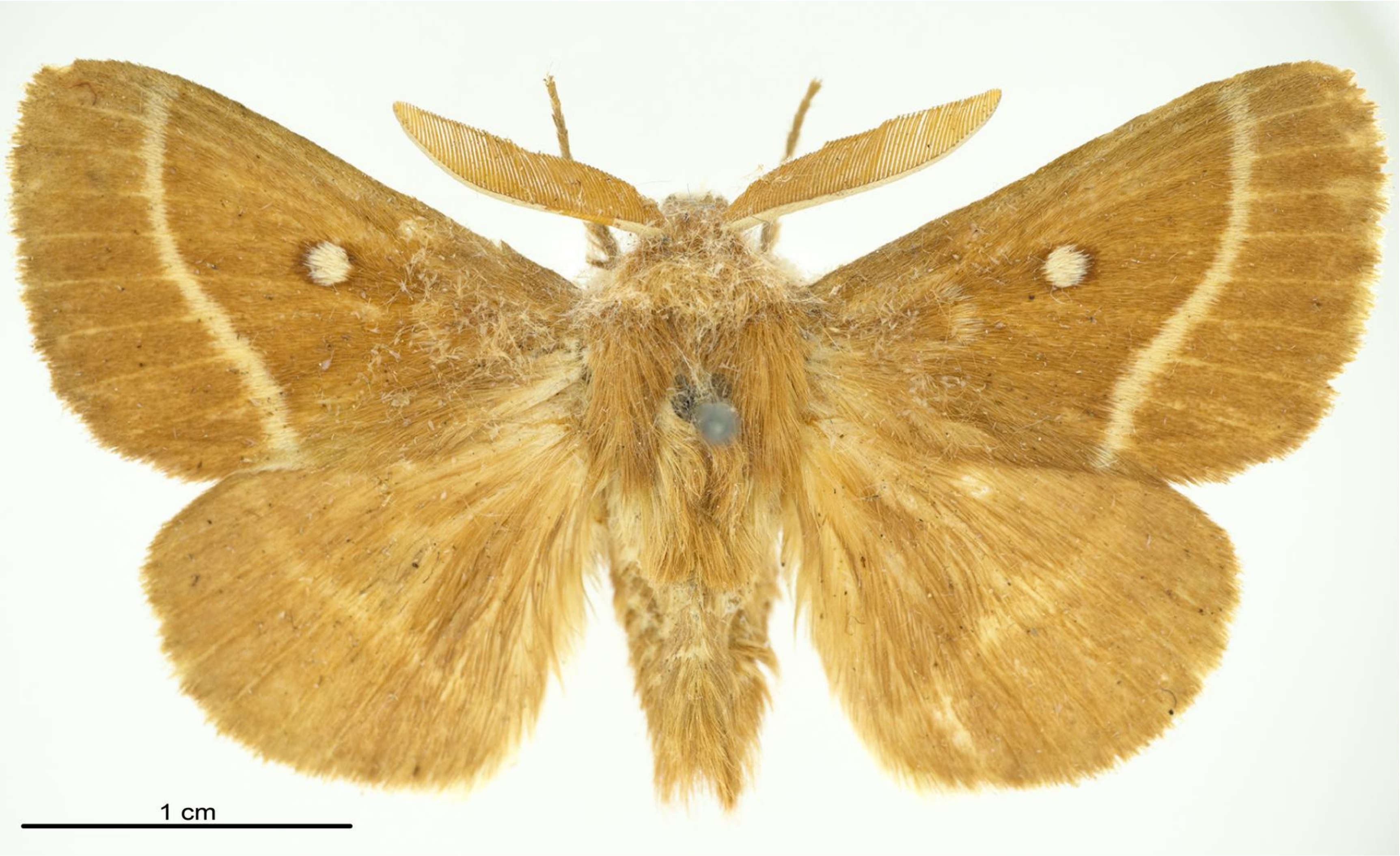

E.8 – *Lassiocampa trifolii*

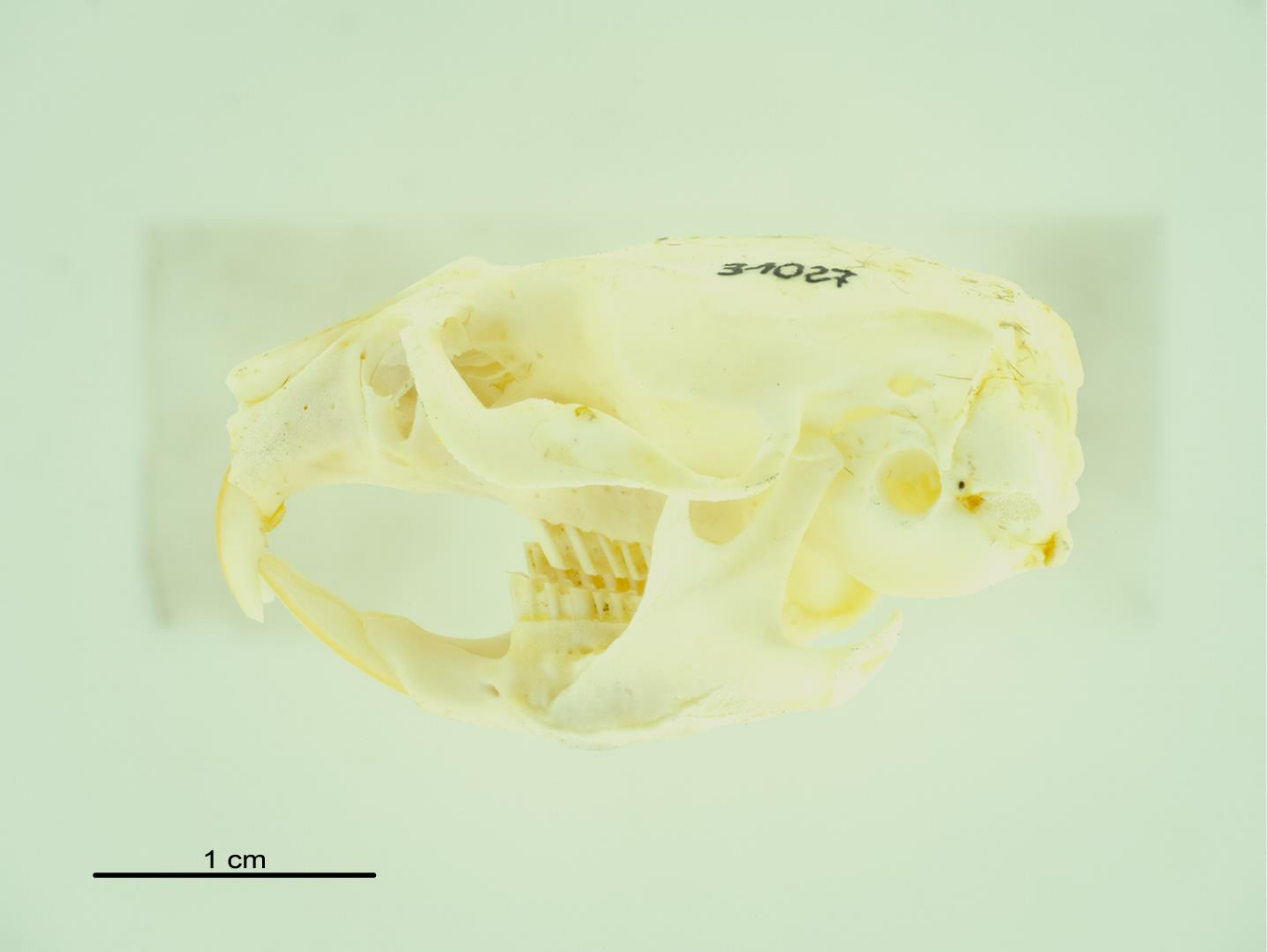

E.9 *Lemmus sibiricus*

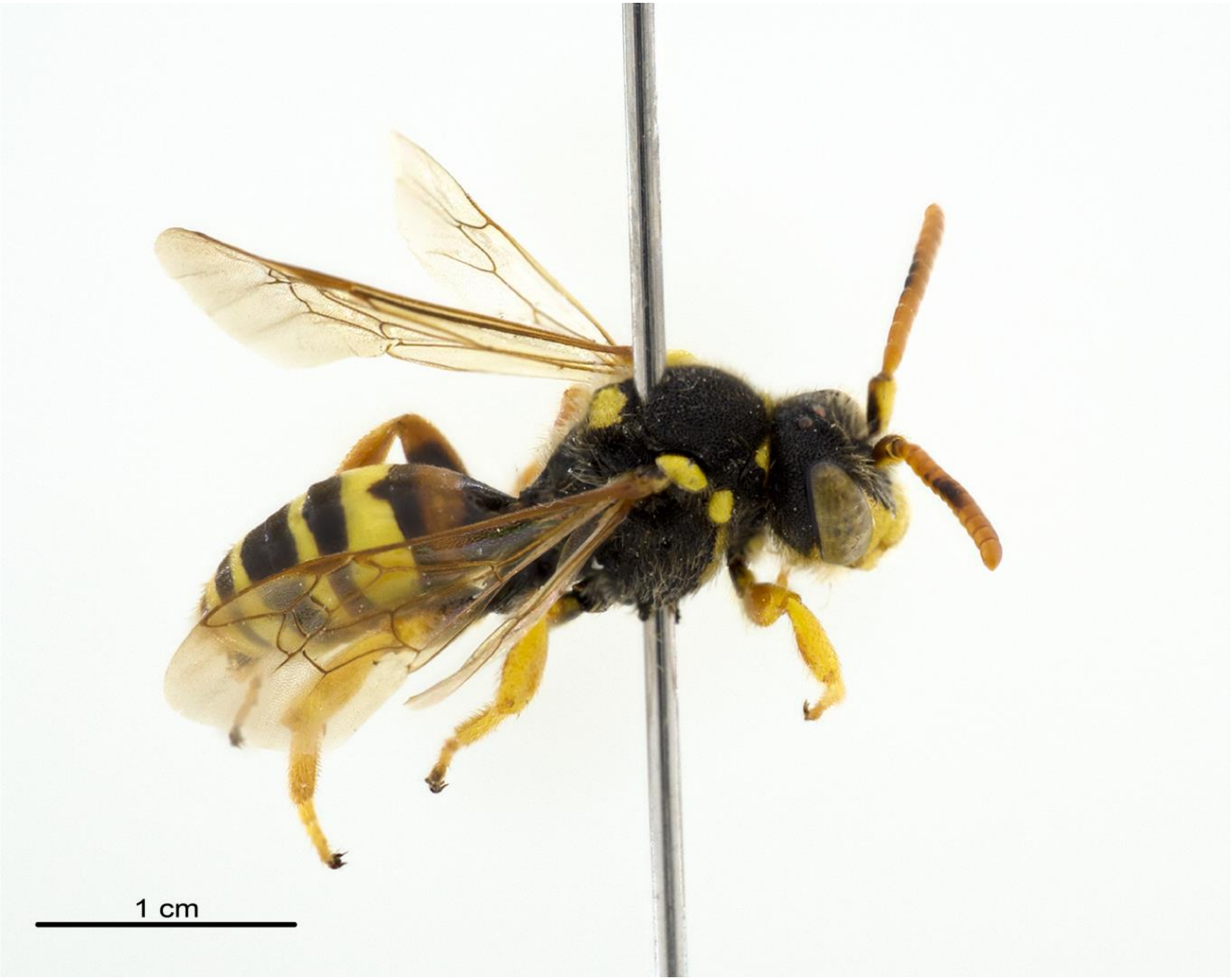

E.10 – *Philanthus Triangulum*

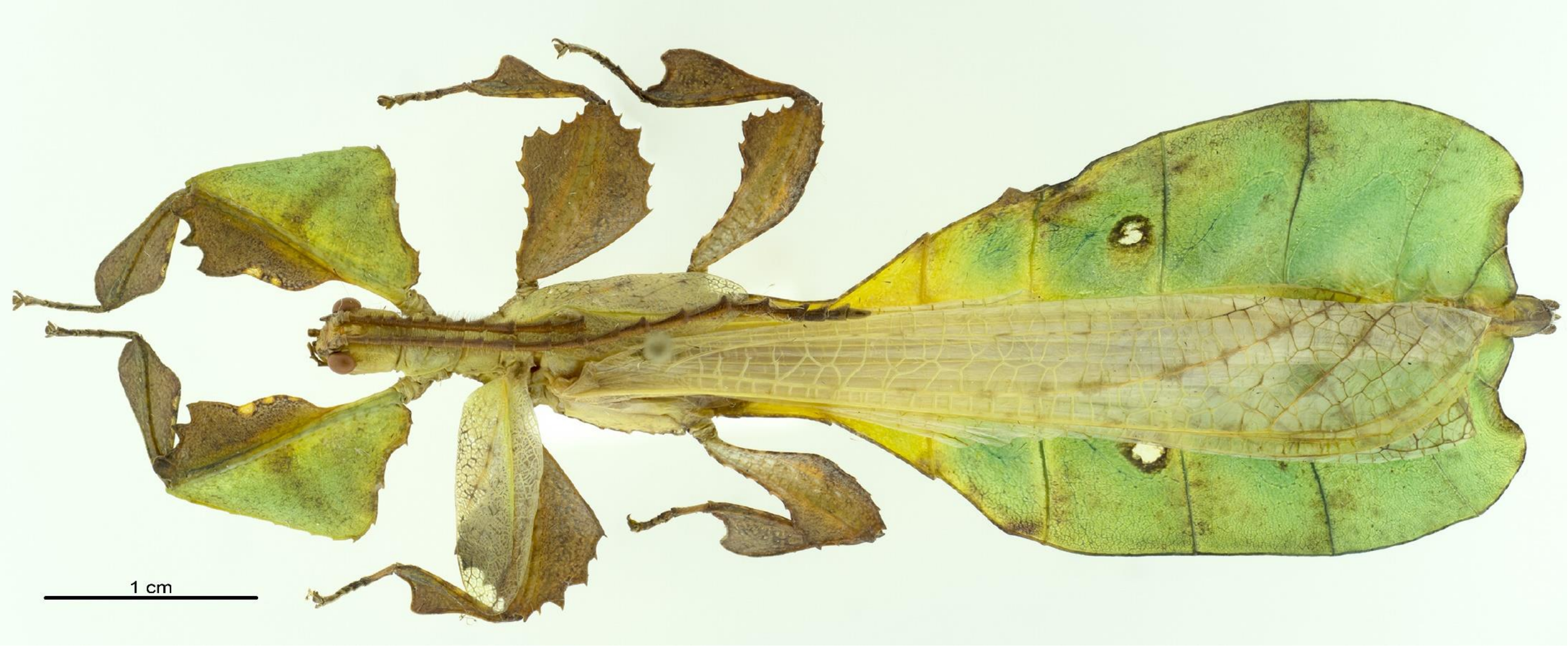

E.11 – *Phyllium pulcrifolium*
